# Impact of the COVID-19 pandemic on diagnosis, and healthcare utilization, among patients with cancer (lung, breast, and pancreas) and cardiovascular diseases (HF, AF, hypertensive, and chronic ischemic heart disease) in Germany: two systematic reviews

**DOI:** 10.1186/s13643-026-03192-z

**Published:** 2026-05-22

**Authors:** Janine Ropeter, Nina Overhageböck, Maren Dreier, Dörthe Meyerdierks, Adil Imran, Torben Heinsohn, Maren Steinmann, Alexander Kuhlmann, Beate Jahn, Berit Lange, Carolina J. Klett-Tammen, Manuela Harries

**Affiliations:** 1 Helmholtz Centre for Infection Research, Department of Epidemiology, Brunswick, Germany; 2 Hannover Medical School, Institute for Epidemiology, Social Medicine and Health Systems Research, Hannover, Germany; 3 School of Public Health, Department of Health Economics and Health Care Management, Bielefeld University, Bielefeld, Germany; 4 Faculty of Medicine, Martin Luther University Halle-Wittenberg, Halle (Saale), Germany; 5 Institute of Public Health, Medical Decision Making and Health Technology Assessment, UMIT TIROL-University for Health Sciences and Technology, Hall I. T, Austria; 6 German Centre for Infection Research (DZIF), TI BBD, Brunswick, Germany

**Keywords:** Germany, Cancer, Cardiovascular diseases, COVID-19, Healthcare utilization, Systematic review

## Abstract

**Background:**

The COVID-19 pandemic and related non-pharmaceutical interventions (NPIs) (e.g., lockdowns and contact restrictions) disrupted routine healthcare delivery. In Germany, these measures affected diagnostic and treatment services for people with cancer and cardiovascular diseases, potentially delaying diagnosis and adversely influencing outcomes. We assessed whether and to what extent diagnosis, health utilization and health outcome among patients with selected cancer and cardiovascular conditions changed in Germany during the pandemic.

**Methods:**

We conducted two systematic reviews of studies from Germany on selected cancers (breast, lung and pancreatic) and cardiovascular conditions (atrial fibrillation/flutter, heart failure, hypertensive and chronic ischemic heart disease). Protocols were registered in PROSPERO and the reviews were reported in accordance with PRISMA. We searched PubMed, Web of Science, Cochrane Library, Scopus, and Embase and screened grey literature. Outcomes included changes in new diagnoses, healthcare utilization, treatment, and disease-specific mortality during the pandemic (2020–2023) compared with the pre-pandemic period (2018–2019). Two reviewers independently screened records, extracted data, and assessed risk of bias using an adapted ROBINS-E tool. Owing to heterogeneity, we synthesized findings narratively.

**Results:**

We screened 1991 records for cancer and 4,981 records for cardiovascular diseases, and included 9 cancer studies and 10 cardiovascular studies. For cancer, several studies reported a relative reduction in new breast and lung cancer diagnoses of up to 25% during lockdown periods; hospital admissions decreased by up to 9%. For cardiovascular conditions, hospital admissions for atrial fibrillation/flutter and heart failure decreased by up to 20%, particularly during pandemic peaks. Evidence on treatment delays, changes in treatment, and mortality was limited, and outcomes for other included diagnoses were often not reported.

**Discussion:**

The available evidence indicates substantial reductions in hospital admissions and new diagnoses among patients with cancer and cardiovascular disease in Germany during the pandemic, suggesting major disruptions to care delivery. However, heterogeneity and gaps in the evidence base limit a comprehensive assessment of downstream outcomes. More comprehensive, linked data and further research are needed to quantify the full pandemic’s impact and to strengthen health-system resilience for future crises.

**Supplementary Information:**

The online version contains supplementary material available at 10.1186/s13643-026-03192-z.

## Key messages

⚈ Compared to 2019, new diagnoses and hospital admissions for both cancer and heart failure (HF)/atrial fibrillation and flutter (AF) declined during the first year of the COVID-19 pandemic.

⚈ In HF/AF, changes in quality of healthcare, including shorter hospital stays, higher rehospitalization rates, fewer cardiovascular-specific procedures, shorter rehab stays, were reported.

⚈ Overall evidence is limited due to (a) a high risk of bias and (b) no data available on outcomes like mortality in cancer or on treatment delays/modifications in both cancer and HF/AF

**Supplementary Information:**

The online version contains supplementary material available at 10.1186/s13643-026-03192-z.

## Background

Since late 2019, SARS-CoV-2 has rapidly spread from China across the globe within a few months, driven by a highly susceptible population, high transmissibility, short incubation period, and global interconnectedness [1]. In the absence of vaccines, testing capacity and treatments, non-pharmaceutical interventions (NPI) became essential to control the pandemic [2]. In Germany, infection control measures included contact restrictions, social distancing, quarantine, school closures, event bans, mandatory testing before entering certain facilities and face-mask mandates as well as travel restrictions, depending on infection rates [3].

These measures led to postponed elective surgeries, cancelled screenings, delayed and reduced medical service provision, and fewer inpatient treatments [4]. In 2020, global cancer screening and diagnostic procedures decreased by 37.3% [95% CI − 44.9 to − 29.7%], accompanied by a 27.0% [95% CI − 32.2 to − 21.8%] reduction in newly diagnosed cases [5]. Similarly, a pattern was observed in cardiovascular care, hospital admissions and cardiology procedures decreasing by 10–66% across Europe during the first wave of COVID-19, varying by condition and country [6].

According to Federal Health Reporting [7], diseases of the circulatory system (33.9%), followed by malignant neoplasms (22.4%), represent the most common causes of death in Germany. Accurate pandemic modeling relies on real-world data to reflect the broader impacts of control measures [8]. To support model parameterization, data on healthcare use among individuals with cancer or cardiovascular disease during the COVID-19 pandemic are needed. Due to existing data gaps and limited comparability, we conducted two systematic reviews to assess the impact of control measures on cancer and cardiovascular disease (CVD) services and outcomes. These reviews analyzed changes in outpatient and inpatient care for affected adults in Germany during different phases of the pandemic (compared to 2018–2019) considering NPIs and trends in disease incidence and mortality. Due to similarities in study design, target population, and policy implications, we report the results of both reviews in a single synthesis.

The review encompasses studies on the following ICD-10 diagnoses: C25 pancreatic cancer (8.4% of diagnosis-specific deaths; 1.9% of total mortality in Germany), C34 lung cancer (19.5%; 4.8%), C50 breast cancer (8.1%; 1.8%), I48 atrial fibrillation/flutter (6.7%; 2.3%), and I50 heart failure (10.8%; 3.7%). We systematically reviewed the impact of COVID-19 on delays and disruptions in cancer and cardiovascular care, comparing pre-pandemic and pandemic periods across early detection, diagnosis, treatment, and therapy. [7].

## Methods

We carried out two systematic reviews on cancer and cardiovascular diseases within the German healthcare setting. The reviews followed the Preferred Reporting Items for Systematic Reviews and Meta-Analyses (PRISMA) Statement. We registered both protocols at PROSPERO (CRD42024539365, CRD42024557960).

The eligibility criteria, including Population, Exposure, Comparator, Outcome, Timing and Settings (PECOTS), are summarized in Table S1 and described below.

### Population

The review focused on adults (≥ 18 years) with either cancer (referred to as systematic review 1, including C34 lung, C50 breast or C25 pancreas cancer) or cardiovascular diseases (referred to as systematic review 2, including I48 atrial fibrillation/flutter, I50 heart failure, I11 hypertensive heart disease, and I25 chronic ischemic heart disease) in Germany during COVID-19 pandemic (2020–2023) compared to pre-pandemic (2018–2019). The three cancer types were selected to reflect a spectrum of malignancies with established screening programs, heterogeneous prognoses, and high lethality. Other major cancer types (e.g., colorectal cancer) were not included, as the focus was on a targeted but diverse disease selection. Cardiovascular diseases were chosen based on identified research gaps, since other systematic reviews already addressed heart attacks and strokes.

### Exposure and comparator

We defined the COVID-19 pandemic (2020–2023) as the exposure of interest, and the pre-pandemic period (2018–2019) as comparator.

### Outcome

The outcomes of interest included changes in diagnosis (incidence, number of new diagnoses and stage at diagnosis), healthcare utilization (e.g., physician contacts, hospital admissions, emergency visits), treatment changes (e.g., invasive procedures, pharmacological therapy, rehabilitation), treatment delays or cancellations, and disease-specific mortality (Table S1).

### Timing and study settings

We included original (peer-reviewed) research articles published after January 1 st, 2018, in German or English (Table S17). We included studies that were conducted in outpatient or inpatient healthcare facilities in Germany. Non-primary publications such as letters, conference abstracts, guidelines, recommendations, editorials, commentary and other reviews were excluded. Full-text articles excluded after detailed assessment are listed in Tables S18a and S18b.

We conducted searches across five electronic databases: PubMed, Web of Science, Embase, Cochrane Library, and Scopus, for both systematic reviews. Additionally, a search for grey literature was performed at the website of the German public health institute, the Robert Koch-Institute [9]. Systematic searches were conducted 29 January–28 February 2024 for cancer and 27 May 2024 for cardiovascular diseases, performed by the student research assistants (NO, JR) as part of their Master’s theses. At that time, no eligible German studies reported longer-term post-pandemic outcomes. Included studies (published 2020–2023) predominantly covered early-pandemic phases (cancer 01/2020–12/2021; CVD 12/2019–12/2020). Data are therefore presented narratively, highlighting the need for continued long-term monitoring.

Three independent reviewers (NO, JR, AI) performed study selection in two steps using the Covidence program [10]. First, titles and abstracts were screened, followed by a full-text review of potentially eligible references. Disagreements were solved through discussion with senior researchers (MD, CKT, MH) to determine final inclusion or exclusion.

After relevant studies were included, the authors NO, JR, and AI extracted data on characteristics of the publication (journal, year, and author), study populations (age, sex, setting), comparison period, disease group (C34 lung, C50 breast, or C25 pancreas cancer, I48 atrial fibrillation/flutter and I50 heart failure) and relevant outcomes, e.g., change of diagnosis, use of healthcare services and disease-specific mortality. The data were extracted by one reviewer and cross-checked by a second reviewer. In case of missing outcomes, the reviewer contacted the author for additional information.

The ROBINS-E (Risk Of Bias In Non-randomized Studies of Exposures) tool was used for the assessment of the risk of bias [11]. The tool was adapted to the study design present in the included studies. It was not possible to perform risk of bias assessment for domain 4 “Risk of bias due to post-exposure interventions” due to the study design of the included studies. Reviewers (NB, JR, AI) extracted the data independently for each study, and extracted data were cross-checked by a second reviewer.

We aimed to perform quantitative synthesis in a meta-analysis; however, due to substantial heterogeneity in populations, effect estimates, and time frames, this was not feasible. Thus, data are synthesized in narrative and tabular form.

## Results

### Study selection

**Fig. 1:**
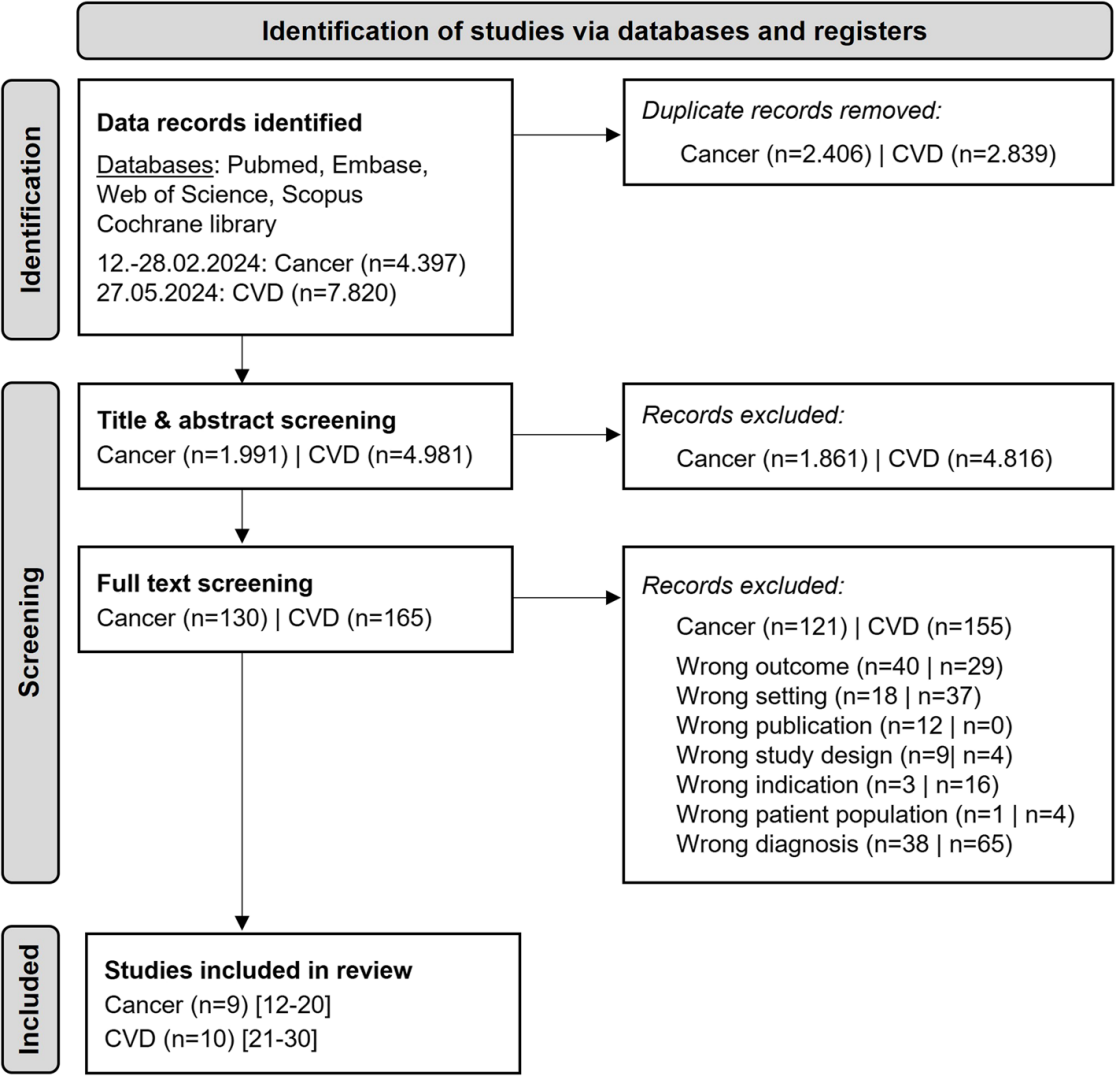
Study-selection flowchart (CVD = cardiovascular diseases)

A total of 4397 studies for cancer (C34 lung, C50 breast, or C25 pancreas cancer) and 7820 for cardiovascular diseases (I48 atrial fibrillation/flutter, I50 heart failure, I11 hypertensive heart disease, I25 chronic ischemic heart disease) were identified through a systematic literature search. Following duplicate removal and title, abstract and full-text screening, 9 studies on cancer and 10 studies on cardiovascular diseases met the inclusion criteria (Fig. 1). No grey literature was identified.

### Study characteristics—cancer

**Fig. 2:**
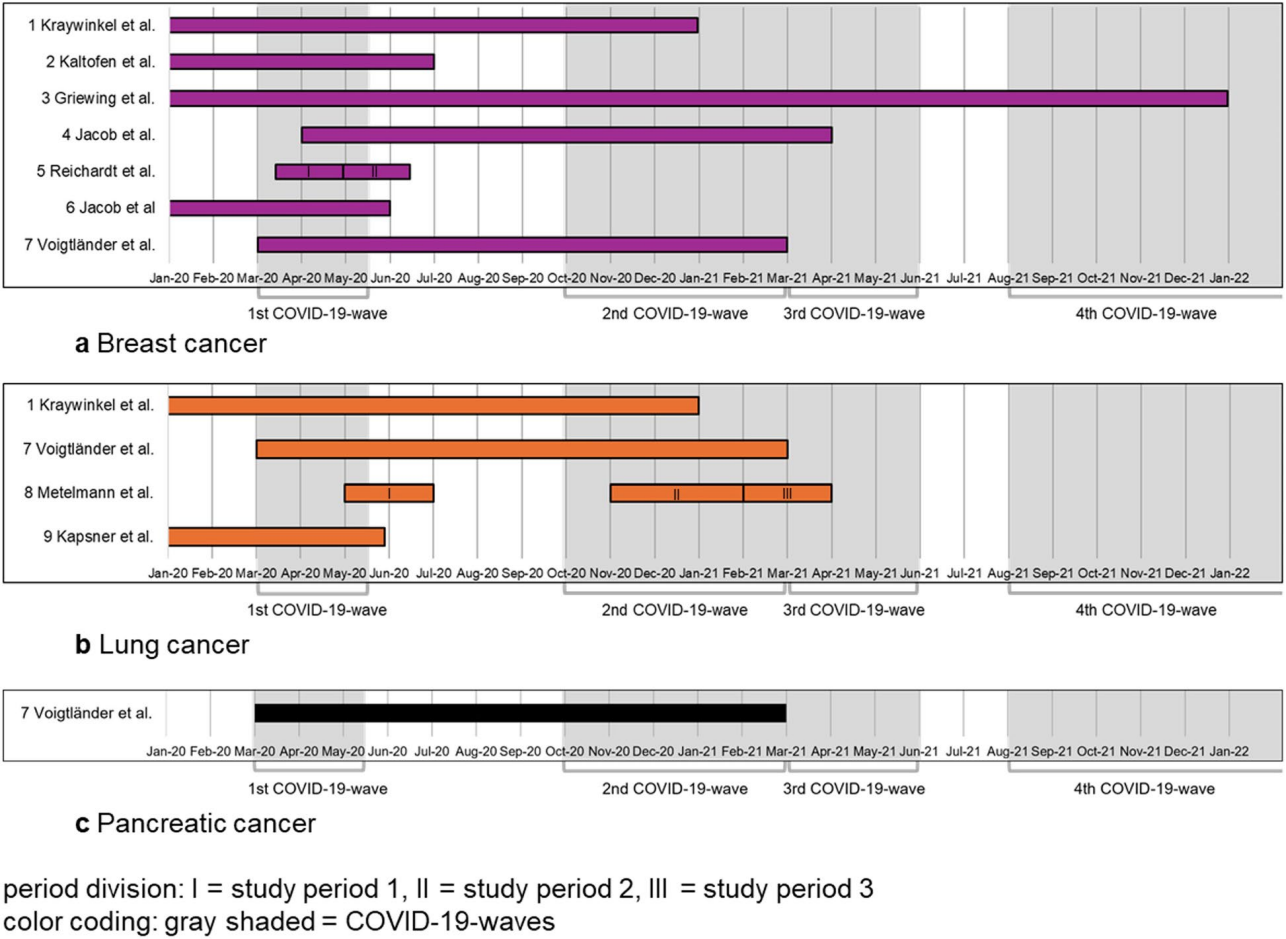
Timeline of study periods for breast (**a**), lung (**b**), and pancreatic cancer (**c**) in 2020–2021

All nine studies applied a before-and-after study design and were published between 2020 and 2023 [11–19]. Four studies focus on hospital settings, using data from the hospital controlling system (2, 3, 5, 9) [12, 13, 15, 19]. The number of hospitals included ranged from one (2, 3) [12, 13] to 75 hospitals (5) [14]. Two studies use data from general and specialized practices (4, 6) [14, 16], and three studies use data from cancer registries (1, 7, 8) [11, 17, 18]. The study periods extend from January 2020 to December 2021 (Fig. 2a–c). The control period varied: the same timeframe in 2019 for most studies, in 2018–2019 in one study, and 2017–2019 in another. Seven studies focus on breast cancer (1–7) [11–17], four on lung cancer (1, 7–9) [11, 17–19], and one on pancreatic cancer (7) [17] (Table 1). Further details on study characteristics are provided in Supplementary Tables S2–S6.

**Table 1:** Study outcomes cancer for breast cancer, lung cancer and pancreatic cancer

	(1)	(2)	(3)	(4)	(5)	(6)	(7)	(8)	(9)	Total		
	Kraywinkel et al. (2023)	Kaltofen et al. (2022)	Griewing et al. (2023)	Jacob et al. (2022)	Reichardt et al. (2021)	Jacob et al. (2021)	Voigtländer et al. (2023)	Metelmann et al. (2023)	Kapsner et al. (2020)			
	[12]	[13]	[14]	[15]	[16]	[17]	[18]	[19]	[20]			
Diagnosis	BC^1^, LC^2^	BC	BC	BC	BC	BC	BC^1^, LC^2^, PC^3^	LC	LC	BC (*n*)	LC (*n*)	PC (*n*)
Incidences and diagnosis stage												
Incidence, new cases	X^1, 2^	X		X		X	X^1, 2, 3^	X		5	3	1
Diagnosis stage	X^1, 2^	X						X		2	2	0
Utilization of healthcare services												
Hospital admissions			X		X				X	2	1	0
Change in treatment										0	0	0
Delays in treatment										0	0	0
Mortality												
Disease specific mortality										0	0	0

^1^*BC* = breast cancer (C50)

^2^*LC* = lung cancer (C34)

^3^*PC* = pancreatic cancer (C25)

### Study population characteristics—cancer patients

One study covers the entire German population (1) [12], two focus on specific federal states (Mecklenburg Western-Pomeranian and Bavaria) (7, 8) [18, 19], and one on the city of Leipzig, Saxony (7) [18]. Four studies examine hospital patients (2, 3, 5, 9) [13, 14, 16, 20] and two studies report outpatient data from a general or specialist practice in Germany (4, 6) [15, 17]. The age is reported in five studies (range 51–69 years) (2, 3, 4, 6, 7) [13–15, 17, 18]. The gender distribution is reported for four studies and varies between 48 and 55% males (3, 4, 6, 7) [14, 15, 17, 18].

### Main reported outcomes—cancer

#### New cancer cases, incidence, and diagnostic stage

**Fig. 3:**
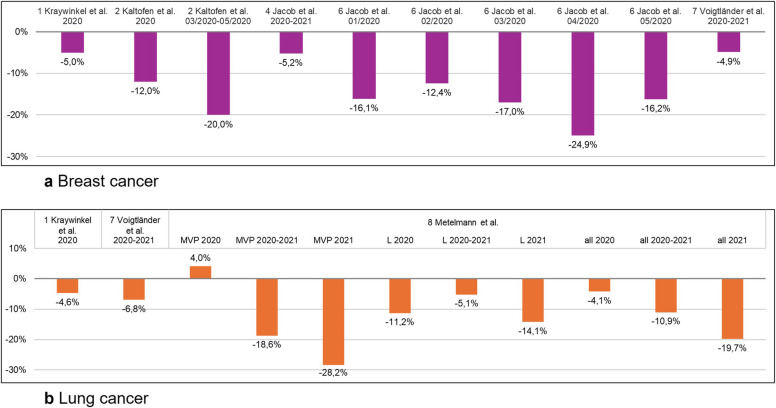
Relative changes in newly diagnosed breast (**a**) and lung cancer (**b**) cases during 2020–2021 compared to control period in Germany

Five studies (studies 1, 2, 4, 6, 7) report a decrease in new cases for breast cancer (Fig. 3a) [12, 13, 15, 17, 18]. Three studies (studies 1, 4, 7) reported a relative decrease in new breast cancer diagnoses of 5.0%, 5.2% and 4.9% (95% CI − 9.3 to − 0.2%) [12, 15, 18]. The other two studies report a higher reduction in new diagnoses. Study 2 shows a relative decline of 12.0% over the whole study period and a relative decline of 20.0% during the lock-down period between March and May 2020 [13]. Study 6 reports monthly decrease between January and May 2020, ranging from − 12.4% in February 2020 to − 24.9% in May 2020 [15]. Study 1, using data from the German cancer registry, found no evidence of a shift toward later tumor stages [12], while study 2 shows a tendency towards a decreased percentage of early TNM (tumor, nodus, metastases) and FIGO (Fédération Internationale de Gynécologie et d'Obstétrique) stages, and an increase in advanced diseases [12].

Studies 1, 7 and 8 report a decrease in new lung cancer diagnoses (Fig. 3b) [12, 18, 19]. Study 1, based on national registry data, shows a relative reduction of new lung cancer diagnoses of 4.6% [12]. Study 7, using data from the Bavarian cancer registry, found a relative decrease of 6.8% (95% CI − 13.6; 0.5) [18]. Study 8, which analyzed data from the cancer registry of Leipzig and Mecklenburg-Western Pomerania, observed a relative increase of 4% in 2020, but a region-specific decline of up to 28.2% in Mecklenburg-Western Pomerania between January and March 2021 [19]. In the same period, a 14.1% decrease was observed in Leipzig [19]. Additionally, study 8 reported an 11.0% decline in stage Ia diagnoses and a 19.0% increase in stage IVb diagnoses [19]. In contrast, study 1 found no shift in tumor stage distribution [12].

Study 7 [18] reports a relative increase of diagnoses for pancreatic cancer by 4.0% (95% CI − 9.4%; 19.4%) (7, pancreatic cancer data not presented in Fig. 3).

### Cancer-related hospital admission

Study 3 [14] shows a 6.0% reduction of hospital admissions for breast cancer in 2020 compared to 2019, with a subsequent increase of 2.0% in 2021. The period was from mid-March to the end of April 2020. Study 5 [16] shows a reduction, compared to the same period in 2019, to 91.0% and, from late April to mid-June, a reduction to 94.0% of the control period.

For lung cancer surgeries, study 9 reports an 8.8% reduction in hospital admissions across 18 university hospitals (9) [20].

No data is available on changes in treatment, treatment delays or disease-specific mortality.

### *Study characteristics*—*CVD*

**Fig. 4:**
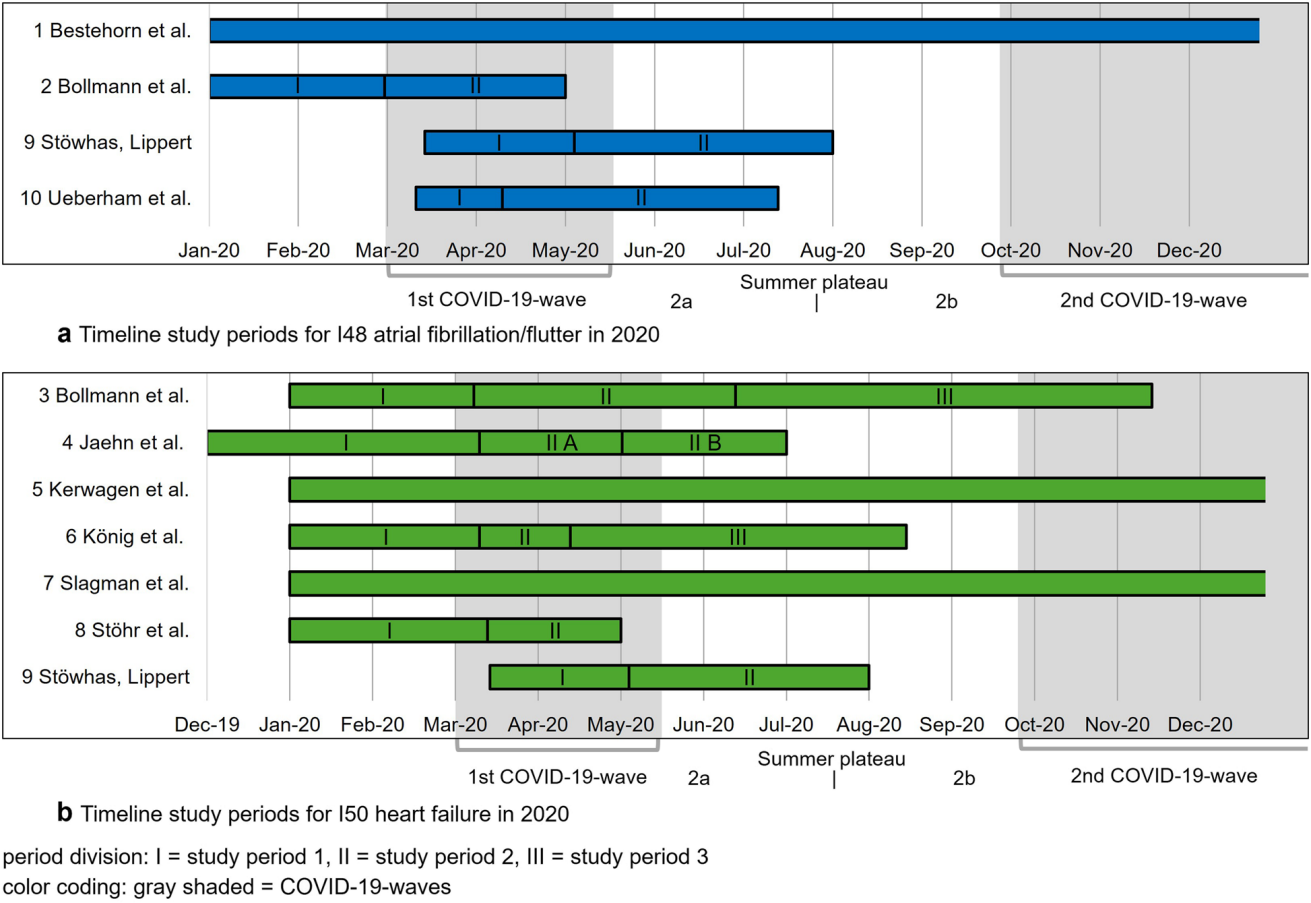
Timeline study periods for I48: atrial fibrillation/flutter (**a**) and I50: heart failure (**b**) in 2020

All studies are before-and-after studies of secondary data and were published between 2020 and 2023. Eight studies (2–4, 6–10) [21–28] focus on hospitals, mostly emergency departments, covering between 1 and 86 hospitals. Study 1 [29] examines 60 cardiac rehabilitation clinics, while study 5 [30] analyzes pharmacy datasets. The exposed study periods extend from 12/2019 to 12/2020 (except study 5: June 2023) and cover the first wave of COVID-19 with 2019 serving as the control period (Fig. 4a, b). Seven studies (2–4, 6, 8–10) reported segmented data to illustrate changes over distinct phases of the pandemic. Details regarding the study and control periods (I–III) for each included study can be found in Supplementary Table S7.

### Characteristics of the study populations—CVD patients

Four studies (1, 2, 9, 10) examine patients with I48 atrial fibrillation/flutter [22, 27–29] and seven with I50 heart failure (3–9) [21, 23–28, 30] (Table 2). Eight studies (2–4, 6–10) examine hospitalized patients [21–28], including seven (2–4, 6–7, 9–10) from emergency departments [21–25, 27, 28]. Study 1 [29] focuses on cardiac rehabilitation, study 5 [30] includes patients with statutory health insurance who were prescribed sacubitril/valsartan (S/V). Three studies report inpatient data only, while five cover both, in- and outpatient care. Study 5 [29] analyzes outpatient prescriptions using pharmacy data, whereas in studies 1 [28] and 8 [25], the care setting, in- or outpatient, remains unclear. The studies focus on populations around retirement age (age range 65–72 years). Age strata vary but show no group differences. Gender distribution is 48.1–53.7% in five studies (4, 6–8, 10) [23–26, 28], while in study 1 [29] 70.8% are men and in study 5 [30] 71.9–81.8%. Three studies report comorbidities (Supplementary Table S9); no other demographic, socioeconomic, psychosocial, or behavioral data are provided. For additional information see Supplementary Table S7.

**Table 2:** Study outcomes CVD for atrial fibrillation/flutter (I48) and heart failure (I50)

	(1)	(2)	(3)	(4)	(5)	(6)	(7)	(8)	(9)	(10)	Total	
	Bestehorn et al. (2022)	Bollmann et al. (2020)	Bollmann et al. (2021)	Jaehn et al. (2021)	Kerwagen et al. (2023)	König et al. (2022)	Slagman et al. (2022)	Stöhr et al. (2020)	Stöwhas, Lippert (2021)	Ueberham et al. (2021)		
	[29]	[22]	[21]	[23]	[30]	[24]	[25]	[26]	[27]	[28]		
Diagnosis	AF^1^	AF	HF^2^	HF	HF	HF	HF	HF	AF, HF	AF	AF (*n*)	HF (*n*)
Incidences and diagnosis stage												
Incidence, new cases											0	0
Diagnosis stage						X				X	1	1
Utilization of healthcare services												
Hospital admissions		X	X	X			X	X	X	X	3	5
Hospital admissions per day		X	X						X		2	2
Duration of hospitalization										X	1	0
Rehospitalizations										X	1	0
Diagnostics and treatment methods										X	1	0
Delays in treatment											0	0
Treatment modification											0	0
Pharmacotherapy					X						0	1
Rehabilitation	X										1	0
Mortality												
Mortality in hospital			X								0	1

^1^*AF* = atrial fibrillation/flutter (I48)

^2^*HF* = heart failure (I50)

### Main outcomes—CVD

#### CVD incidence and diagnosis stages

None of the included studies reported incidence rates or newly diagnosed cases. Study 10 [28] reports the Charlson Comorbidity Index (CCI) and CHA_2_DS_2_-VASc score aggregated for incident and prevalent hospital admissions due to I48 atrial fibrillation/flutter. The CCI measures the impact of comorbidities on mortality [31], while the CHA_2_DS_2_-VASc score assesses stroke risk [32]. Higher scores indicate increased risk. The CCI distribution shows no significant differences (*p* > 0.05), with 68.0% of participants achieving a score of 0–1. The CHA_2_DS_2_-VASc score indicates a significant before-and-after shift (*p* < 0.01) from 2–4 points (− 2.8%) to ≥ 5 points (+ 2.1%) indicating an increased stroke risk during the pandemic. Study 6 [24] analyzes New York Heart Association (NYHA) classes II–IV for severity of heart failure (I50). The absolute number of patients decreases in all classes. Compared to the control period, relative changes towards more severe cases are observed: NYHA II-16.3%, NYHA III+2.4%, and NYHA IV+1.4% (Supplementary Table S10).

### Hospital admissions

**Fig. 5:**
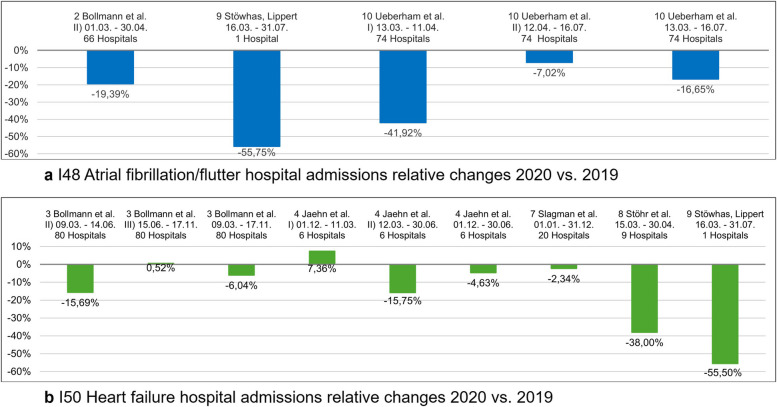
I48 atrial fibrillation/flutter (**a**) and I50 heart failure (**b**) hospital admissions relative changes 2020 Δ 2019 in Germany

The absolute number of hospital admissions for atrial fibrillation/flutter (I48) and heart failure (I50) decreased across all studies and phases (Supplementary Table S10). The relative changes are shown in Fig. 5 for I48 (5a) and for I50 (5b). Study 10 [28] shows a higher relative reduction for I48 in prevalent cases (−19.0% [95% CI − 21.0; − 17.0%]) than in incident cases (− 14.0% [95% CI − 16.0; − 12.0%]). Prevalent cases were defined as those with prior admissions within the past 3 years, while incident cases had none. Study 10 [28] further finds a greater decline in regular admissions (21.6%) vs. emergency admissions (12.0%) for I48, with similar distribution across periods. Study 9 [27] finds differences in self-presentations vs. specialist referrals for I48 and I50. For I48, self-presentations have a lower reduction (47.2%) than referrals (59.7%), while for I50, self-presentations have a higher reduction (62.8%) than referrals (52.9%). Study 4 [23] splits period II into IIA (March 12–May 6, 2020) and IIB (May 7–June 30, 2020) and calculates adjusted rate ratios (RR) for I50, controlling for age group, sex, and hospital in the comparison of hospital admission rates. IIA shows an RR of 0.7 [95%-CI 0.7; 0.8] and IIB shows an RR of 0.97 [95% CI 0.85; 1.10]. Study 7 [25] also shows that in 2020, outpatient emergency visits (− 17.3%) declined more than inpatient admissions (− 6.9%) across all diagnoses (I50 heart failure, I21 myocardial infarction, I20, angina pectoris, R07 chest pain).

### Hospital admissions per day

Study 3 [21] analyzes daily hospital admissions for heart failure (I50) using rate ratios. Period II (March 9–June 14, 2020) shows a significantly lower risk (RR = 0.84, 95%-CI 0.81–0.87; *p* < 0.01), while period III (June 15–November 17, 2020 vs. 2019) shows no difference (RR = 1.01, 95% CI 0.98–1.03). Study 2 [22] applies the same method for I48 atrial fibrillation/flutter, finding a significantly lower risk in period II (March 1–April 30, 2020) compared to the previous year (RR = 0.74, 95% CI 0.68–0.79) and compared to period I (January 1–February 28, 2020) (RR = 0.81, 95% CI 0.75–0.87; *p *< 0.01). Study 9 [27] reports a reduction in daily hospital admissions of 18.18% for I48 (29.55% in period I (March 16–May 06, 2020 vs. 2019), 9.09% in period II (May 7–July 31, 2020 vs. 2019)) and 16.22% for I50 (17.57% in period I, 16.22% in period II). During period I, I48 self-presentations increased by 24%, while referrals decreased by 55%. In period II, self-presentations decreased by 17% and referrals by 7%. For I50, self-presentations declined by 32% in period I and 30% in period II, with referrals decreasing by 12% in both periods (Supplementary Table S11).

### Duration of hospitalization

Study 10 [28] reports an average stay of 3.3 ± 3.1 nights (study group) vs. 3.5 ± 3.6 nights (control group) for I48, with a regression coefficient of −0.23 (95% CI − 0.35, − 0.11).

### Rehospitalization

Study 10 [28] reported rehospitalization rates for I48 atrial fibrillation/flutter at 21.7% (*n* = 1519) for the study group vs. 23.6% (*n* = 1988) in the control group. This corresponds to a relative reduction of 23.6% during the study period.

### Diagnostic and treatment interventions

No study reported invasive examinations. Study 10 [28] determined the number of semi-invasive transesophageal echocardiographies (TEE), non-invasive electrical cardioversions (ECV) and minimally invasive catheter ablations (CA) for the diagnosis of I48 atrial fibrillation/flutter. All procedures decreased during the study period. In period I, TEEs decreased by 6.7% in absolute terms and by −16.2% relative to the reference period (OR 0.74 [95% CI 0.64; 0.86]). Results for TEE in period II, ECV, and CA were not statistically significant (Supplementary Table S12).

### Pharmacotherapy

Study 5 [30] examined COVID-19’s impact on guideline-directed therapies for I50 heart failure with reduced ejection fraction (HFrEF), focusing on sacubitril/valsartan including prescriptions of sodium-glucose transporter 2 inhibitors (SGLT2i) between 2018 and 2023. The study refers to the “Guidelines for the diagnosis and treatment of acute and chronic heart failure” of the European Society of Cardiology from 2021 and analyzes the prescription of S/V and the combination SGLT2i from 2018 to 2023. The S/V market grew from 310,778 (2018) to 1,176,368 (2022) patients. Prescriptions increased, especially for S/V + SGLT2i. At the pandemic’s onset, growth slowed in Q2 (+ 8.96%) and Q3 (+ 6.85%) of 2020 compared to Q1 (+ 13.56%) in 2020.

### Rehabilitation of CVD patients

Study 1 [29] shows a 32.5% decline in cardiac rehabilitation for I48 atrial fibrillation/flutter during the COVID-19 pandemic, with an average duration of 30.9 weeks (46.7% reporting a decrease for the whole of 2020). A total of 22% of rehabilitation clinics repurposed beds for COVID-19, while 42.4% had outbreaks, causing 15.7% partial closures. Rehabilitation recorded decreases in follow-up treatment (− 14.2%), medical rehab (− 16.7%) and aftercare procedures (− 36.6%). All rehabilitation measures declined, with the largest decreases in doctor’s rounds (− 44.6% total interventions | − 35.4% interventions per person) and training kitchens (− 44.6% | − 32.5% p.p.). In contrast, physiotherapy (− 11.1% |+ 8.2% p. p.), occupational therapy (− 13.2% |+ 5.2% p. p.), social counseling (− 13.5% |+ 5.0% p. p.), nutritional counseling (− 15.0% |+ 3.3% p. p.), and nursing visits (− 15.5% |+ 2.3% p. p.) were less affected, with some even showing an increase in the changes per person (Supplementary Table S14).

### CVD-related mortality

Study 5 [30] reports on in-hospital mortality for heart failure (I50). In-hospital mortality was 6.9% in the study group for period I (03/20–06/20) compared to 5.6% in the control group (OR = 1.26 [95% CI 1.08; 1.47]). In period II (06/20–11/20), it was 5.7% vs. 5.2% (OR = 1.11 [95% CI 0.97; 1.27]).

### Assessment of risk of bias in cancer and CVD studies

**Fig. 6:**
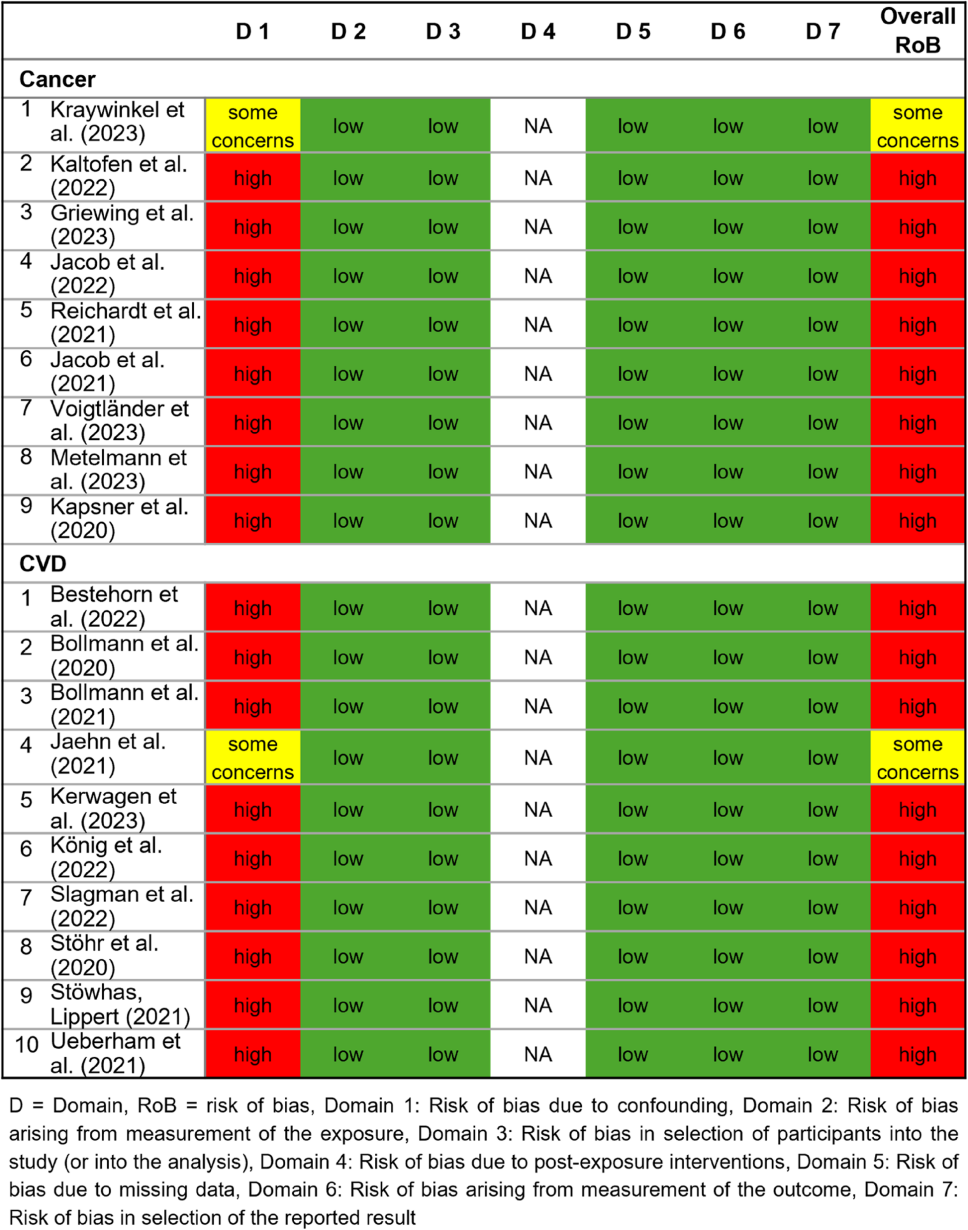
Results of the risk-of-bias assessment ROBINS-E

Seventeen of 19 studies included were rated as having a “high risk of bias” (ROBINS-E [11]), primarily due to confounding (Domain 1, Fig. 6). None of the studies adjusted for all key confounders. In uncontrolled before-and-after study designs, minimizing bias requires that the characteristics of the population and the healthcare facility remain stable between the pre- and post-intervention periods. Important potential confounders include seasonality, population characteristics (e.g., age distribution, proportion of migrants, comorbidities), and structural features of healthcare facilities (e.g., staffing levels and qualifications, service capacity, and clinical specialties).

Two studies by Kraywinkel et al. (2023) [12] and Jaehn et al. (2021) [23] were rated as having “some concern” in Domain 1, because they accounted for selected key confounders. All other domains were rated as “low risk of bias” and Domain 4 (post-exposure interventions) was considered “not applicable”.

## Discussion

This review provides an overview of healthcare utilization among patients with cancer or cardiovascular diseases in Germany across different phases of the COVID-19 pandemic. The included 19 before-and-after studies differ in observation periods, study designs, sample sizes, care settings (inpatient vs. outpatient), geographic scopes (national vs. local, urban vs. rural) and patient characteristics (e.g., age, comorbidities).

The narrow temporal focus on early pandemic phases reflects the scope of the German evidence available at the time the searches were conducted, rather than a limitation of the review design. Included studies (published 2020–2023) covered the following periods: cancer 01/2020–12/2021 and CVD 12/2019–12/2020. No eligible studies addressing longer-term post-pandemic outcomes were identified, emphasizing the need for continued monitoring.

Across both, cancer (lung, breast, and pancreas) and cardiovascular diseases (heart failure, atrial fibrillation), a marked decline in hospital admissions was observed during the pandemic compared to the pre-pandemic period: ranging from 6 to 8.8% for cancer and 16.6 to 55.7% for cardiovascular diseases (CVD), particularly during the early pandemic phase, with partial recovery in 2021. However, evidence on treatment delays, changes in care modalities, and disease-specific outcomes remains limited, indicating substantial knowledge gaps.

The decline in new cancer diagnoses during the COVID-19 pandemic appears multifactorial. Key contributors include healthcare system constraints (e.g., prioritization of COVID-19 patients, suspension of non-urgent care) and reduced access to routine screenings [33]. In parallel, patient hesitancy regarding healthcare access due to fear of infection further reduced early detection opportunities [4]. Psychological and behavioral factors influencing patient decisions were not captured in the included studies and represent a limitation of this review; these aspects should be explored in future research.

Breast cancer diagnoses declined by approximately 5% in several studies (cancer nos. 1, 4, and 7) [12, 15, 18], with more pronounced drops during the first lockdown, up to 20% in some regions (cancer nos. 2 and 6) [13, 17]. This aligns with national data demonstrating an 8.1% reduction in mammography screenings in 2020, with temporary suspension of screening programs [33], and a 31% decrease in screening diagnoses among BARMER-insured patients [23]. Some patients reportedly skipped medical appointments despite experiencing symptoms [4]. These disruptions likely contributed to a stage shift in cancer diagnoses, with fewer early-stage and more advanced-stage cancers detected (cancer no. 2) [13]. Similar patterns were reported for lung cancer, with new diagnoses decreasing by 4.6% and 6.8%, and local reports indicating up to 28.2%, often accompanied by evidence of stage migration indicative of diagnostic delays (cancer nos. 1, 7, and 8) [12, 18, 19]. However, not all studies confirmed stage shift (cancer no. 1) [12], pointing to regional or methodological variation. In contrast, pancreatic cancer diagnoses slightly increased, possibly due to its aggressive nature, which might lead to more urgent initiation of diagnosis regardless of pandemic conditions. Registry reporting delays may also have contributed to reduced diagnosis rates in some datasets [12].

The data from the German cancer registry at the Robert Koch-Institute supports these findings, revealing an initial decline in breast, lung, and pancreatic cancer diagnoses in 2020, followed by a partial rebound in 2021 and slight decreases in 2022 [20]. These fluctuations likely reflect diagnostic delays rather than an actual reduction in cancer incidence. The temporary suspension of screening programs and patients’ avoidance of healthcare services led to underdiagnosis, consistent with a 22% drop in neoplasm-related hospital admissions in early 2020 [4]. Registry reporting delays may further complicate trend interpretation [12].

Evidence on cancer treatment during the pandemic is sparse, but disruptions in hospital-based cancer care are evident. Breast cancer admissions declined by 6% in 2020 [15], with some recovery in 2021. Lung cancer surgeries dropped by 8.8% across several hospitals [14], aligning with the 22% drop in cancer-related hospital admissions reported nationally by the Robert Koch-Institute [4]. No data were available for pancreatic cancer admissions.

Seven included studies reported a decline in CVD hospital admissions in Germany (CVD nos. 2–4 and 7–10) [21–23, 25–28]. The most pronounced reductions occurred during the first COVID-19 wave, with admissions for I48 atrial fibrillation/flutter decreasing by 16.65 to 55.75% and admissions for I50 heart failure by 15.59 to 38.00%. Emergency visits declined slightly less among self-presenting patients (47.22%) than among those referred by physicians (52.86%) (CVD no. 9) [27], although it remains unclear whether this reflects changes in healthcare-seeking behavior, reduced access to general practitioners, or both. One study reported a particularly marked decline in inpatient admissions (CVD no. 10) [28], which is plausible given the prioritization of urgent treatments during the pandemic [4]. The same study also found shorter lengths of stay and fewer rehospitalizations, potentially helping to preserve capacity for COVID-19 care. However, an international review found no consistent evidence for changes in length of stay among CVD patients [34].

These findings are consistent with the 2024 German Heart Report [35] which identified the steepest decline during the first COVID-19 wave followed by a modest rebound, and with Khan et al. (2023) [6], who reported a 9–66% reduction in heart failure admissions across Europe between 2020 and 2022. The observed reductions are clinically relevant, particularly in the context of constrained ICU capacity in 2020 and 2021.

 [35]. However, reduced hospital utilization should not be interpreted as a lower underlying disease burden. Delayed care may have contributed to disease progression or avoidable mortality [36]. while the extent to which outpatient services compensated remains uncertain. More granular, day-specific analyses are needed to assess temporal variation and the impact of non-pharmaceutical interventions.

Despite overall declines in inpatient care, Kerwagen et al. (2023) [27] reported continued increases in prescriptions for heart failure therapies (e.g., sacubitril/valsartan and SGLT2 inhibitors), with slower growth during the first COVID-19 wave. The 2024 German Heart Report [35] confirms this trend, with notable increases in SGLT2i prescriptions, especially in 2022 [35] after the publication of the European Society of Cardiology’s 2021 guideline. In 2023, SGLT2i have also been approved for new indications [37, 38], which may have further driven this trend. Overall, pharmaceutical supply appeared stable.

Evidence from rehabilitation settings was addressed by one study. Bestehorn et al. (2022) [29] (CVD no. 1) reported a 32.5% relative decline in cardiac rehabilitation among patients with atrial fibrillation in 2020, especially among retirees. Reductions mainly affected medical visits and group sessions, while individual treatments increased. Reasons included fear of infection, NPIs, capacity management, and temporary closures. These findings align with the global impact on cardiac rehabilitation [39]. Compared to Nadarajah et al. (2022) [40], Germany had relatively few program closures, while more virtual consultations occurred internationally, such as mostly home-based rehab in the UK (OR _2020 Δ 2019_ = 59.2) [41]. In Germany, digital services remain rare outside of aftercare [42]. Long-term effects of missed rehab remain unknown.

The only study we identified reporting mortality was conducted by Bollmann et al. (2021) [21] (CVD no. 3) and showed a 26% increased hospital mortality for heart failure during the first COVID-19 wave (03–06/2020) compared to the previous year. Overall, mortality was slightly higher in 2020 but declined later in the year. In Europe, in-hospital mortality during the pandemic was 6–7% until 2022 compared to pre-pandemic mortality of 5–6% [36]. An international review shows an increase in the relative risk of dying from heart failure to 1.1 by the end of 2021, with moderate heterogeneity (*I*^2^ = 63.9%) [40]. This may reflect the overload of the healthcare system or treatment delays. According to Schäfer et al. (2023) [43] healthcare providers attributed non-utilization to political restrictions (10.8%), infected staff (8.1%), and capacity preservation (6.8%), while patients most often reported fear of infection (42.2%) and waiting for safer conditions (12.9%).

## Limitations

Few studies met the inclusion criteria, and heterogeneity in periods, settings, and outcomes precluded meta-analysis and limited external validity. The variability in study periods limits the conclusive statements regarding the impact of non-pharmaceutical intervention. Many studies had a high risk of bias due to insufficient control for confounders, limited statistical reporting, or lack of group comparisons. Moreover, the focus on 2020–2021 excludes later pandemic phases and long-term impacts. These limitations reflect the German evidence available at the time of our searches (cancer January–February 2024; CVD May 2024) and could not be influenced by this review, highlighting the need for future studies with more complete outcome data.

## Recommendation for future research

Based on the gaps identified in this review, future research should assess longer-term post-pandemic outcomes and later phases of health crises, with improved control for key confounders such as age, comorbidities, and care setting. Linking outpatient, hospital, and registry data would provide a more comprehensive view of care pathways and should include outcomes such as treatment delays, therapy changes, rehospitalizations, and disease-specific mortality, particularly among vulnerable groups. In addition, digital real-time reporting systems, closer collaboration with health insurance providers, and faster data-sharing and publication processes would enhance timely monitoring and support more agile, evidence-informed public health decision-making during future emergencies.

## Conclusions

This synthesis reveals a measurable impact of the COVID-19 pandemic on healthcare utilization among patients with cardiovascular and oncological conditions in Germany, especially during the initial phase. Declines in diagnoses, hospital admissions, and treatment were consistently observed. While limitations in data granularity, study periods, and settings persist, the trends are robust and reliable, particularly due to the inclusion of registry data providing population-level insights.

To support future decision models on non-pharmaceutical interventions, more detailed and linked data across outpatient, hospital, and registry settings are needed. Despite current gaps, particularly in long-term outcomes and outpatient care, the existing evidence is sufficient to identify critical disruptions and inform public health planning.

## Supplementary Information

Supplementary Material 1.

## Data Availability

According to our FAIR principle, data are available on request.

## Abbreviations

AF: Atrial fibrillation/atrial flutter
BC: Breast cancer
CA: Catheter ablations
CCI: Charlson Comorbidity Index
CVD: Cardiovascular diseases
ECV: Electrical cardioversions
FIGO: Fédération Internationale de Gynécologie et d'Obstétrique
HF: Heart failure
HFrEF: Heart failure with reduced ejection fraction
LC: Lung cancer
NPI: Non-pharmaceutical interventions
PC: Pancreatic cancer
PRISMA: Preferred Reporting Items for Systematic reviews and Meta-Analysis
ROBINS-E: Risk of bias in non-randomized follow-up studies of exposure effects
SGLT2i: Sodium glucose transporter 2 inhibitors
S/V: Sacubitril/valsartan
TEE: Transesophageal echocardiographies
TNM: Tumor, nodus, metastases
