# Supplementary material for: Impact of the COVID-19 pandemic on diagnosis, and healthcare utilization, among patients with cancer (lung, breast, and pancreas) and cardiovascular diseases (HF, AF, hypertensive, and chronic ischemic heart disease) in Germany: two systematic reviews
Source: Syst Rev. 2026 May 22;15:172. doi: 10.1186/s13643-026-03192-z (PMC13196194; doi:10.1186/s13643-026-03192-z)
Supplement: Supplementary file 1 — Supplementary Material 1. [file 13643_2026_3192_MOESM1_ESM.docx]

**Supplementary**

[Table S 1: PRISMA Checklist 2](#_Toc227571778)

[Table S 2: Inclusion and exclusion criteria 6](#_Toc227571779)

[Table S 3: Cancer study characteristics 7](#_Toc227571780)

[Table S 4: Cancer study population characteristics 9](#_Toc227571781)

[Table S 5: Number of new diagnoses for cancer 10](#_Toc227571782)

[Table S 6: Diagnosis stages for cancer 11](#_Toc227571783)

[Table S 7: Number of & change in hospital admissions for cancer 12](#_Toc227571784)

[Table S 8: CVD study characteristics 13](#_Toc227571785)

[Table S 9: Table S8 CVD study population characteristics 15](#_Toc227571786)

[Table S 10: Comorbidities of cardiovascular patients 17](#_Toc227571787)

[Table S 11: Diagnosis stages: distribution of NYHA classes, CCI & CHA2DS2 VASc scores in 2020 18](#_Toc227571788)

[Table S 12: Number of & change in hospital admissions for cardiovascular diseases in 2020 19](#_Toc227571789)

[Table S 13: Number of diagnostics and treatment for I48 atrial fibrillation/flutter 20](#_Toc227571790)

[Table S 14: Number of S/V prescriptions 2018 - 2023 (Kerwagen et al., 2023, p. 4f.) 21](#_Toc227571791)

[Table S 15: Measures in cardiac rehabilitation in 2020 (Bestehorn et al., 2022, p. 25) 22](#_Toc227571792)

[Table S 16: Cancer search string 23](#_Toc227571793)

[Table S 17: CVD search string 27](#_Toc227571794)

[Table S 18: Search result for cancer - deduplication with Endnote & Covidence 32](#_Toc227571795)

[Table S 19: Search result for CVD - deduplication with Endnote & Covidence 33](#_Toc227571796)

[Table S 20: Excluded studies from the full text screening cancer (breast, lung, pancreatitis) 34](#_Toc227571797)

[Table S 21: Excluded studies from the full text screening cancer (breast, lung, pancreatitis) 39](#_Toc227571798)

Table S 1: PRISMA Checklist

| **Section and Topic** | **Item #** | **Checklist item** | **Location where item is reported** |
| --- | --- | --- | --- |
| **TITLE** | | |  |
| Title | 1 | Identify the report as a systematic review. | P1 line1-2 |
| **ABSTRACT** | | |  |
| Abstract | 2 | See the PRISMA 2020 for Abstracts checklist. | P2 line 32-62 |
| **INTRODUCTION** | | |  |
| Rationale | 3 | Describe the rationale for the review in the context of existing knowledge. | P5 line 75- 99 |
| Objectives | 4 | Provide an explicit statement of the objective(s) or question(s) the review addresses. | P6 line 100- 105 |
| **METHODS** | | |  |
| Eligibility criteria | 5 | Specify the inclusion and exclusion criteria for the review and how studies were grouped for the syntheses. | P6 line 112- 129, Supplement table S1 |
| Information sources | 6 | Specify all databases, registers, websites, organisations, reference lists and other sources searched or consulted to identify studies. Specify the date when each source was last searched or consulted. | P7 line 130- 133 |
| Search strategy | 7 | Present the full search strategies for all databases, registers and websites, including any filters and limits used. | P7 line 130-133, Supplement table 15-16 |
| Selection process | 8 | Specify the methods used to decide whether a study met the inclusion criteria of the review, including how many reviewers screened each record and each report retrieved, whether they worked independently, and if applicable, details of automation tools used in the process. | P7 line 135- 137, Supplement table S1 |
| Data collection process | 9 | Specify the methods used to collect data from reports, including how many reviewers collected data from each report, whether they worked independently, any processes for obtaining or confirming data from study investigators, and if applicable, details of automation tools used in the process. | P7-8 line 138-144 |
| Data items | 10a | List and define all outcomes for which data were sought. Specify whether all results that were compatible with each outcome domain in each study were sought (e.g. for all measures, time points, analyses), and if not, the methods used to decide which results to collect. | P7-8 line 138-142 |
|  | 10b | List and define all other variables for which data were sought (e.g. participant and intervention characteristics, funding sources). Describe any assumptions made about any missing or unclear information. | P7-8 line 138-144 |
| Study risk of bias assessment | 11 | Specify the methods used to assess risk of bias in the included studies, including details of the tool(s) used, how many reviewers assessed each study and whether they worked independently, and if applicable, details of automation tools used in the process. | P8 line 145-150 |
| Effect measures | 12 | Specify for each outcome the effect measure(s) (e.g. risk ratio, mean difference) used in the synthesis or presentation of results. | Supplement Tables S4-S14 |
| Synthesis methods | 13a | Describe the processes used to decide which studies were eligible for each synthesis (e.g. tabulating the study intervention characteristics and comparing against the planned groups for each synthesis (item #5)). | P8 line 151-153 |
|  | 13b | Describe any methods required to prepare the data for presentation or synthesis, such as handling of missing summary statistics, or data conversions. | P8 line 151-153 |
|  | 13c | Describe any methods used to tabulate or visually display results of individual studies and syntheses. | P8 line 151-154 |
|  | 13d | Describe any methods used to synthesize results and provide a rationale for the choice(s). If meta-analysis was performed, describe the model(s), method(s) to identify the presence and extent of statistical heterogeneity, and software package(s) used. | P8 line 151-154 |
|  | 13e | Describe any methods used to explore possible causes of heterogeneity among study results (e.g. subgroup analysis, meta-regression). | P8 line 151-154, P25 449-454 limitations |
|  | 13f | Describe any sensitivity analyses conducted to assess robustness of the synthesized results. | P8 line 151-154 |
| Reporting bias assessment | 14 | Describe any methods used to assess risk of bias due to missing results in a synthesis (arising from reporting biases). | P8 line 146-150 |
| Certainty assessment | 15 | Describe any methods used to assess certainty (or confidence) in the body of evidence for an outcome. | P8 line 146-150 |
| **RESULTS** | | |  |
| Study selection | 16a | Describe the results of the search and selection process, from the number of records identified in the search to the number of studies included in the review, ideally using a flow diagram. | P9 line 155-161, Fig 1 |
|  | 16b | Cite studies that might appear to meet the inclusion criteria, but which were excluded, and explain why they were excluded. | P9 Fig. 1 |
| Study characteristics | 17 | Cite each included study and present its characteristics. | P10 line 166-187 and P14 line 228-256 |
| Risk of bias in studies | 18 | Present assessments of risk of bias for each included study. | P20 line 348-355 |
| Results of individual studies | 19 | For all outcomes, present, for each study: (a) summary statistics for each group (where appropriate) and (b) an effect estimate and its precision (e.g. confidence/credible interval), ideally using structured tables or plots. | P12 line 192-227 and P16 line 257-347 |
| Results of syntheses | 20a | For each synthesis, briefly summarise the characteristics and risk of bias among contributing studies. | P21,line 350-354, Fig 6 |
|  | 20b | Present results of all statistical syntheses conducted. If meta-analysis was done, present for each the summary estimate and its precision (e.g. confidence/credible interval) and measures of statistical heterogeneity. If comparing groups, describe the direction of the effect. | P11-13, p16-20 |
|  | 20c | Present results of all investigations of possible causes of heterogeneity among study results. | P8 line 152-154 |
|  | 20d | Present results of all sensitivity analyses conducted to assess the robustness of the synthesized results. |  |
| Reporting biases | 21 | Present assessments of risk of bias due to missing results (arising from reporting biases) for each synthesis assessed. | P20 line 348-355 |
| Certainty of evidence | 22 | Present assessments of certainty (or confidence) in the body of evidence for each outcome assessed. | P21 Fig. 6 |
| **DISCUSSION** | | |  |
| Discussion | 23a | Provide a general interpretation of the results in the context of other evidence. | P22 line 356-446 |
|  | 23b | Discuss any limitations of the evidence included in the review. | P25 line 447-453 |
|  | 23c | Discuss any limitations of the review processes used. | P25 line 447-453 |
|  | 23d | Discuss implications of the results for practice, policy, and future research. | P26 line 454-464 |
| **OTHER INFORMATION** | | |  |
| Registration and protocol | 24a | Provide registration information for the review, including register name and registration number, or state that the review was not registered. | P6 line 110-111 |
|  | 24b | Indicate where the review protocol can be accessed, or state that a protocol was not prepared. | P6 line 111-112 |
|  | 24c | Describe and explain any amendments to information provided at registration or in the protocol. | P6 line 111-112 |
| Support | 25 | Describe sources of financial or non-financial support for the review, and the role of the funders or sponsors in the review. | P28 line 493-495 |
| Competing interests | 26 | Declare any competing interests of review authors. | P27 line 488 |
| Availability of data, code and other materials | 27 | Report which of the following are publicly available and where they can be found: template data collection forms; data extracted from included studies; data used for all analyses; analytic code; any other materials used in the review. | P28 line 500-501, Supplement |

This work is licensed under CC BY 4.0. To view a copy of this license, visit https://creativecommons.org/licenses/by/4.0/

Table S 2: Inclusion and exclusion criteria

|  | **Inclusion criteria** | **Exclusion criteria** |
| --- | --- | --- |
| **Population** | Adulte people with cancer / CVD during the COVID-19 pandemic | People without cancer / CVD  Children (<18 years)  People with COVID-19 infection |
| **Exposure** | COVID-19 pandemic |  |
| **Control** | Adulte people with cancer / CVD before the COVID-19 pandemic (2018-2019) | People without cancer / CVD  Children (<18 years) |
| **Outcome** | diagnosis (incidence, number of new diagnoses and stage at diagnosis)  healthcare utilization, (e.g. physician contacts, hospital admissions, emergency visits)  change of treatment (e.g. invasive procedures, pharmacological therapy, rehabilitation)  treatment delays or cancellations  disease specific mortality | Screenings  Treatment due to coronavirus infection/ vaccination |
| **Setting** | Outpatient and inpatient healthcare facilities in Germany:  Physicians practice  Clinics  Hospitals  Rehabilitation centers |  |
| **Study design** | (Controlled) before-and-after study  Interrupted time series study  Randomized controlled trial  Cross-sectional study  Case-control study  Cohort study  Longitudinal study  Case report  Qualitative study  Ecological study | Reviews  Intervention studies |
| **Type of publication** | Peer-reviewed publications | Conference abstracts  Letter  Guidelines  Commentary  Recommendations  Editorials |
| **Publication date** | Cancer: Since 1st January 2018  CVD: 01.01.2018 – 27.05.2024 | Cancer. Before 1st January 2018  CVD: before 01.01.2018, after 27.05.2024 |
| **Language** | German  English | Other languages |
| **Country** | Germany | Other countries |

Table S 3: Cancer study characteristics

| **No.** | **Authors** | **Year** | **Region** | **Setting** | **Data source** | **Study**  **period** | **Study duration** | **Control**  **period** | **Control duration** |
| --- | --- | --- | --- | --- | --- | --- | --- | --- | --- |
| **1** | Kraywinkel  et al. (2023) | 2023 | DE | N/A | German wide data of the Zentrum für Krebsregisterdaten at RKI | 01.01.2020  – 31.12.2020 | 366 Days | 01.01.2019  – 31.12.2019 | 365 Days |
| **2** | Kaltofen  et al. (2022) | 2022 | Munich | LMU Munich | Data is based on the specification of the national federation commission of the German Cancer Society | I) 01.01.2020  – 30.06.2020  II) 22.03.2020  – 05.05.2020 | 182 Days  45 Days | I) 01.01.2019  – 30.06.2019  II) 22.03.2019  – 05.05.2019 | 181 Days  45 Days |
| **3** | Griewing  et al. (2023) | 2023 | Marburg | German university maximum care provider/ Marburg University Hospital | Data from hospital controlling program | 01.01.2020  – 31.12.2021 | 731Days | 01.01.2017  – 31.12.2019 | 1095 Days |
| **4** | Jacob  et al. (2022) | 2022 | DE | general and specialized practices | Data from Disease Analyzer Database (IQVIA) | 01.04.2020  – 31.03.2021 | 366 Days | 01.04.2019  – 31.03.2020 | 365 Days |
| **5** | Reichardt  et al. (2021) | 2021 | DE | 75 Helios Hospitals in Germany | Routine data from hospitals | I)  13.03.2020  – 28.04.2020  II) 29.04.2020  – 14.06.2020 | 47 Days  47 Days | I)  13.03.2019  – 28.04.2019  II) 29.04.2019  – 14.06.2019 | 47 Days  47 Days |
| **6** | Jacob  et al. (2021) | 2021 | DE | general and specialized practices | Data from Disease Analyzer Database (IQVIA) | 01.01.2020  – 31.05.2020 | 152 Days | 01.01.2019  – 31.05.2019 | 151 Days |
| **7** | Voigtländer  et al. (2023) | 2023 | Bavaria | N/A | Data from Bavarian Cancer Registry | 01.03.2020  – 28.02.2021 | 365 Days | 01.03.2019  – 28.02.2020 | 365 Days |
| **8** | Metelmann  et al. (2023) | 2023 | Leipzig  MWP | N/A | Data from cancer registries of Leipzig and Mecklenburg-Western Pomeranian | I)  01.05.2020  – 30.06.2020  II) 01.11.2020  – 31.01.2021  III) 01.02.2021  – 31.03.2021 | 61 Days  92 Days  59 Days | I)  01.05.2019  – 30.06.2019  II) 01.11.2019  – 31.01.2020  III) 01.02.2020  – 31.03.2020 | 61 Days  92 Days  60 Days |
| **9** | Kapsner  et al. (2020) | 2020 | DE | 18 University Hospitals | Claim data | 01.01.2020  – 31.05.2020 | 152 Days | I)   01.01.2018  – 31.05.2018  II)  01.01.2019  – 31.05.2019 | 151 Days  151 Days |

N/A = not available, BC = breast cancer, LC = lung cancer

Table S 4: Cancer study population characteristics

| **No.** | **Diagnosis** | **Population** | **Sample size study (n)** | **Sample size control (n)** | **Age study**  (median1 \| mean, SD2)  [years] | **Age control** (mean, SD \| %, IQR)  [years] | **Sex study**  (% male, IQR) | **Sex control**  (% male, IQR) |
| --- | --- | --- | --- | --- | --- | --- | --- | --- |
| 1 | BC  LC | residents of Germany | N/A | N/A | N/A | N/A | N/A | N/A |
| 2 | BC | patients at LMU | 327 | 365 | 54.71  March-May: 50,81 | 56.61  March-May: 57,81 | N/A | N/A |
| 3 | BC | hospitalized patients at Marburg University Hospital | 991 | 1.432 | female: 63,22  male: 622 | | 54.5% | |
| 4 | BC | patients ≥ 18 years with at least 1 visit at 1 of 1403 general or specialized practices in Germany | 126.379 | 138.996 | 65.9(16.0)2 | 65.6 (15.9)2 | 49.3% | 49.2% |
| 5 | BC | patients at one of the 75 Helios Hospitals in Germany | I) 345.7 admissions/day   II) 332.1 admissions/day | I) 400.3 admissions/day  II) 386.9 admissions/day | N/A | N/A | N/A | N/A |
| 6 | BC | patients ≥ 18 years with at least 1 visit at 1 of 1660 general or specialized practices in Germany | 47.142 | 54.867 | 64.7 (16,2)2 | 64.4 (16,2)2 | 49.6% | 48.0% |
| 7 | BC  LC  PC | residents of Bavaria | 39980 | 42857 | male: 67.8 (12.7)2  female: 64.0 (15.4)2  male: 691  female: 651 | | 50.0% | |
| 8 | LC | residents of Mecklenburg-Western Pomeranian and Leipzig | 3372 (2019-2021)  Leipzig: 1598 (2019-2021)  MWP: 1774 (2019-2021) | | N/A | N/A | N/A | N/A |
| 9 | LC | patients with inpatient admission at one of 18 University hospitals | N/A | N/A | N/A | N/A | N/A | N/A |

N/A = not available, BC = breast cancer, LC = lung cancer

Table S 5: Number of new diagnoses for cancer

| **No.** | **Diagnosis** | **Number of new cancer diagnoses** | | |
| --- | --- | --- | --- | --- |
|  |  | **Study group**  (n^1^ \| mean per practice^2^) | **Control group**  (n^1^ \| mean per practice^2^) | **Relative change (95% CI)** |
| 1 | BC  LC | N/A | N/A | BC:  all: -5.0%  female: -5.0%  0-49 years: -3.1%  50-69 years: -6.1%  70- years: -4.4% |
|  |  |  |  | LC:  all: -4.6%  female: -4.3%  male: -4.7%  0-49 years: -10.6%  50-69 years: -4.4%  70- years: -4.4% |
| 2 | BC | I) 1501  II) 24^1^ | I) 170^1^  II) 30^1^ | I) -12%  II) -20% |
| 4 | BC | 10.8 (SD 20,2)^2^ | 10.2 (SD 16,3)^2^ | -5.2% (p=0.674) |
| 6 | BC | Jan: 2.8 (SD 3.2)^2^  Feb: 2.5 (SD 2.0)^2^  Mar: 2.4 (SD 2.1)^2^  Apr: 2.1 (SD 1.6)^2^  May: 2.3 (SD 2.7)^2^ | Jan: 3.4 (SD 5.9)^2^  Feb: 2.8 (SD 4.4)^2^  Mar: 2.9 (SD 5.0)^2^  Apr: 2.9 (SD 4.3)^2^  May: 2.8 (SD 4.6)^2^ | Jan: -16.1%  Feb: -12.4%  Mar: -17.0%  Apr: -24.9%  May: -16.2% |
| 7 | BC | 8278^1^ | 8703^1^ | -4.9% (-9.3; -0.2) |
|  | LC | 3294^1^ | 3536^1^ | -6.8% (-13.6; 0.5) |
|  | PC | 1039^1^ | 999^1^ | +4.0% (-9.4; 19.4) |
| 8 | LC | I) 257  II) 309  III) 208 | I) 268  II) 347  III) 259 | I) -4.1%  II) -10.9%  III) -19.7% |
|  |  | Leipzig  I) 130  II) 122  III) 74 | I) 125  II) 150  III) 103 | I) +4%  II) -18,6%  III) -28,2% |
|  |  | MWP  I) 127  II) 187  III) 134 | I) 143  II) 197  III) 156 | I) -11,2%  II) -5,1%  III) -14,1% |

N/A = not available, BC = breast cancer, LC = lung cancer, PC =pancreatic cancer

Table S 6: Diagnosis stages for cancer

| **Kaltofen et al. (2022)** | | | **Metelmann et al. (2023)** | |  |
| --- | --- | --- | --- | --- | --- |
| **BC** | | | **LC** | |  |
| Study  Tis: 12 (7%)  T1: 53 (31%)  T2-4: (27%)  N+: (24%)  M1: (11%) | Control  Tis: 11 (7%)  T1: 46 (31%)  T2-4: 30 (20%)  N+: 46 (31%)  M1: 17 (11%) | Change  Tis: 0  T1: -1  T2-4: -7  N+: 7  M1: 0 | Relative change  Period II:  Stage Ia: -10%  Period III:  Stage IVb: +19%  Stage Ia: -11% | p-value  Period II:  Stage Ia significant decrease (p=0.012)  Period III:  stage IVb (p=0.007)  stage Ia (p=0.049) |  |

N/A = not available, BC = breast cancer, LC = lung cancer

Table S 7: Number of & change in hospital admissions for cancer

|  |  |  | **Number of hospital admissions** | | | **Number of hospital admissions per day** | | |  |
| --- | --- | --- | --- | --- | --- | --- | --- | --- | --- |
| **No.** | **Publication** |  | **Study group** | **Control group** | **Relative change (95% CI)** | **Study group** | **Control group** | **Relative change (95% CI)** |  |
| 3 | Griewing  et al. (2023) | BC | 2020: 475  2021: 516 | 2017: 453  2018: 473  2019: 506 | 2017 to 2018: +4%  2018 to 2019: +7%  2019 to2020: -6%  2019 to 2021: +2% | N/A | N/A | N/A |  |
| 5 | Reichardt  et al. (2021) | BC | N/A | N/A | N/A | I) 48.2  II) 44.1 | I) 53.0  II) 47.2 | I) 0.91 (0.86-0.96)  II) 0.94 (0.88-0.99) |  |
| 9 | Kapsner  et al. (2020) | LC | 93 | 102 | -8.8% | N/A | N/A | N/A |  |

N/A = not available, BC = breast cancer, LC = lung cancer

Table S 8: CVD study characteristics

| No. | Authors | Year | Region | Setting | Data source | Data collection | Study  period | Study duration | Control  period | Control duration |
| --- | --- | --- | --- | --- | --- | --- | --- | --- | --- | --- |
| 1 | Bestehorn  et al. | 2022 | DE | 60 DGPR-Rehabilitation clinics | 2 Online surveys at DGPR member hospitals | 2020  05/21 -11/21  2019  05/20 - 11/20 | 01.01.2020  – 31.12.2020 | 366 Days | 01.01.2019  – 31.12.2019 | 365 Days |
| 2 | Bollmann  et al. | 2020 | DE | 66 Helios clinics | Accounting data | N/A | I) 01.01.2020  – 28.02.2020  II) 01.03.2020  – 30.04.2020 | 59 Days  61 Days | 01.03.2019  – 30.04.2019 | 61 Days |
| 3 | Bollmann  et al. | 2021 | DE | 80 Helios clinics | QlikView® | N/A | I) 01.01.2020  – 17.11.2020  II) 09.03.2020  – 14.06.2020  III) 15.06.2020  – 17.11.2020 | 322 Days  98 Days  156 Days | I) 01.01.2019  – 17.11.2019  II) 11.03.2019  − 16.06.2019  III) 17.06.2019  − 17.11.2019 | 321 Days  98 Days  154 Days |
| 4 | Jaehn et al. | 2021 | 5 cities  in DE | 6 Hospital accident & emergency departments  (standard & maximum care) | Routine data from the accident & emergency departments | N/A | I) 02.12.2019  – 11.03.2020  II) 12.03.2020  – 30.06.2020  IIA) 12.03.2020 – 06.05.2020  IIB) 07.05.2020 – 30.06.2020 | 101 Days  111 Days  56 Days  55 Days | I) 01.12.2018  – 11.03.2019  II) 12.03.2019  – 30.06.2019  IIA) 12.03.2019 – 06.05.2019  IIB) 07.05.2019  – 30.06.2019 | 101 Days  111 Days  56 Days  55 Days |
| 5 | Kerwagen et al. | 2023 | DE | Pharmacies | IQVIA Data set | N/A | 01.01.2020  – 30.06.2023 | 182 Days | 01.01.2016  – 31.12.2019 | 1461 Days |
| 6 | König et al. | 2022 | DE | 86 Helios clinics | Hospital routine data | N/A | I) 01.01.2020  – 11.03.2020  II) 12.03.2020  – 14.04.2020  III) 15.04.2020  – 18.08.2020 | 73 Days  34 Days  126 Days | I) 01.01.2019  – 12.03.2019  II) 13.03.2019  – 15.04.2019  III) 16.04.2019  – 19.08.2019 | 73 Days  34 Days  126 Days |
| 7 | Slagman  et al. | 2022 | DE | 20 Hospital emergency rooms (university & non-university) | EDIS & AKTIN-  Emergency room register | 01/21 - 02/21 | 01.01.2020  – 31.12.2020 | 366 Days | 01.01.2019  – 31.12.2019 | 365 Days |
| 8 | Stöhr et al. | 2020 | NW:  2x Bonn,  7x Rhein-Sieg-Kreis | 9 Hospital emergency rooms | Rescue service & Emergency doctor reports | N/A | I) 01.01.2020  – 14.03.2020  (excl. 29.02.)  II) 15.03.2020  – 30.04.2020 | 75 Days  47 Days | I) 01.01.2019  – 14.03.2019  II) 15.03.2019  – 30.04.2019 | 75 Days  47 Days |
| 9 | Stöwhas,  Lippert | 2021 | MV:  Güstrow | 1 Hospital emergency room | Routine data from the emergency room | N/A | I) 16.03.2020  – 06.05.2020  II) 07.05.2020  – 31.07.2020 | 52 Days  86 Days | 03.07.2019  – 15.03.2020 | 257 Days |
| 10 | Ueberham et al. | 2021 | DE  (13/16  Federal states) | 74 Hospitals | QlikView® | N/A | I) 13.03.2020  – 11.04.2020  II) 12.04.2020 – 16.07.2020 | 30 Days  96 Days | I) 15.03.2019  – 13.04.2019  II) 14.04.2019  – 18.07.2019 | 30 Days  96 Days |
| N/A = not available, DE = Germany, NW = North Rhine Westphalia, MV = Mecklenburg-Western Pomerania, I/ II/ III = data set is divided into study-specific time periods | | | | | | | | | | |

Table S 9: Table S8 CVD study population characteristics

| **Study No.** | **Diagnosis** | **Population** | **Sample size study (n)** | **Sample size control (n)** | **Age study**  (mean, SD \| %, IQR)  [years] | **Age control** (mean, SD \| %, IQR)  [years] | | **Sex study**  (% male, IQR) | **Sex control**  (% male, IQR) | |
| --- | --- | --- | --- | --- | --- | --- | --- | --- | --- | --- |
| 1 | I48 | Cardiac rehabilitation patients | 96 | 142 | 65,2 ± 5.1^1^ | 65.7 ± 5.0^1^ | | 70.79%^1^ | 70.06%^1^ | |
| 2 | I48 | Inpatient emergency room patients | I) 1,739  II) 1,322 | II) 1,640 | N/A | N/A | | N/A | N/A | |
| 3 | I50 | Inpatient emergency room patients | 14,288  I) N/A  II) 5,187  III) 9,101 | 15,206  I) N/A  II) 6,152  III) 9,054 | N/A | N/A | | N/A | N/A | |
| 4 | I50 | Outpatient & inpatient emergency room patients | 1,938  I) 1,050  II) 888 | 2,032  I) 978  II) 1,054 | I) ^1^ \| II) ^1^  18-64: 53.7% \| 54.0%  > 64: 42.6% \| 41.7% | I) ^1^ \| II) ^1^  18-64: 54.5% \| 55.3%  > 64: 41.9% \| 39.6% | | I) 50.1%^1^  II) 51.0%^1^ | I) 49.9%^1^  II) 50.5%^1^ | |
| 5 | I50 | 80% of SHI-insured persons with  a prescription (outpatient) for S/V | 3,499,937  2020: 720,364  Q1: 165,676  Q2: 174,480  Q3: 184,947  Q4: 195,261  2021: 914,480  Q1: 207,290  Q2: 219,762  Q3: 325,367  Q4: 252,061  2022: 1,176,368  Q1: 270,326  Q2: 286,160  Q3: 301,287  Q4: 318,595  2023: 688,725  Q1: 337,463  Q2: 351,262 | 822,140  2018: 310,778  Q1: 63,632  Q2: 72,704  Q3: 81,644  Q4: 92,798  2019: 511,362  Q1: 107,069  Q2 121,044  Q3: 134,080  Q4: 149,169 | >80:  S/V \| S/V+SGLT2i  2020:  Q1: 27.4% \| 8.7%  Q2: 27.8% \| 9.2%  Q3: 28.1% \| 9.8%  Q4: 28.3% \| 11.0%  2021:  Q1: 29.1% \| 12.0%  Q2: 30.1% \| 13.3%  Q3: 30.8% \| 15.2%  Q4: 31.8% \| 18.0%  2022:  Q1: 32.9% \| 19.5%  Q2: 33.8% \| 21.2%  Q3: 34.7% \| 22.4%  Q4: 35.2% \| 23.2%  2023:  Q1: 35.8% \| 24.1%  Q2: 36.2% \| 24.8%  Q2/2023:^1^  >80: 29.9%  71-80: declined  <60: declined | >80:  S/V \| S/V+SGLT2i  2018:  Q1: 23.6% \| 7.4%  Q2: 24.2% \| 7.0%  Q3: 24.1% \| 7.0%  Q4: 24.5% \| 6.9%  2019:  Q1: 25.4% \| 7.0%  Q2: 26.0% \| 7.6%  Q3: 26.4% \| 8.1%  Q4: 26.9% \| 8.5% | | S/V \| S/V+SGLT2i  2020:  Q1: 68.7% \| 81.3%  Q2: 68.7% \| 81.4%  Q3: 68.6% \| 81.8%  Q4: 68.1% \| 81.1%  2021:  Q1: 68.1% \| 79.8%  Q2: 67.8% \| 78.4%  Q3: 67.5% \| 77.5%  Q4: 66.9% \| 76.0%  2022:  Q1: 66.3% \| 74.9%  Q2: 65.5% \| 73.9%  Q3: 65.2% \| 73.4%  Q4: 64.8% \| 72.9%  2023:  Q1: 64.3% \| 72.3%  Q2: 64.0% \| 71.9% | S/V \| S/V+SGLT2i  2018:  Q1: 70.0% \| 80.9%  Q2: 69.9% \| 81.4%  Q3: 69.7% \| 81.5%  Q4: 69.7% \| 82.5%  2019:  Q1: 69.7% \| 82.5%  Q2: 69.4% \| 81.6%  Q3: 69.1% \| 81.5%  Q4: 69.1% \| 81.6% | |
| 6 | I50 | Inpatients with NYHA class I-IV | 12,781 | 13,526 | predicted^1^ \| observed^1^  <65: 12.6% \| 11.0%  65-74: 16.3% \| 17.6%  >75: 72.7% \| 69.8% | < 65: 11.5%^1^  65-74: 17.5%^1^  > 75: 71.1%^1^ | | predicted: 48.9%^1^  observed: 48.1%^1^ | 49.0%^1^ | |
| 7 | I50 | emergency room patients | 13,164 | 13,480 | < 60: 55.79%^1.2^  (31.79%-75.66%)^1^  ≥ 60: 39.40%^1.2^  (24.67%-49.34%)^1^ | < 60: 58.5%^1.2^  (31.84%-78.67%)^1^  ≥ 60: 38.04%^1.2^  (22.33%-46.97%)^1^ | | 52.65%^1,2^  (29.64%-66.23%)^1^ | 52.88%^1,2^  (27.09%-63.98%)^1^ | |
| 8 | I50 | emergency room patients | 489 | N/A | 71.9 ± 15.1^1^ | | 49.7%^1^ | | |  |
| 9 | I48,  I50 | emergency room patients Güstrow | I48: 50  I50: 85 | I48: 113  I50: 191 | I) ^1^ \| II) ^1^  18-49: 28% \| 26%  50-69: 27% \| 29%  70-89: 29% \| 28%  >90: 3% \| 3% | 18-49: 28%^1^  50-69: 25%^1^  70-89: 26%^1^  >90: 2%^1^ | |  |  | |
| 10 | I48 | Outpatient & inpatient emergency room patients | 7,001  I) 1,331  II) 5,670 | 8,424  I) 2,326  II) 6,098 | 70.3 ± 11.7 | 70.1 ± 11.8 | | 53.7% | 54.4% | |
| N/A = not available, SD = standard deviation, IQR = interquartile range, ^1^ = data for the entire study, ^2^ = data for median CW, I/ II/ III = data set is divided into study-specific time periods | | | | | | | | | | |

Table S 10: Comorbidities of cardiovascular patients

|  | 1 Bestehorn et al. (2022)^1^  study \| control | 6 König et al. (2022)^1^  study \| control | 10 Ueberham et al. (2021)  study \| control |
| --- | --- | --- | --- |
| Main diagnosis | **I48 Atrial fibrillation/ flutter** | **I50 Heart failure** | **I48 Atrial fibrillation/ flutter** |
| Neoplasms |  |  | any malignancy: 0.7% \| 0.7%  metastatic solid tumour: 0.1% \| 0.2% |
| Nutrition & Metabolism | diabetes melitus: 20.9% \| 20.1%  lipid metabolism disorder: 43.1% \| 44.5%  adiposity / obesity: 15.7% \| 15.2% | diabetes melitus  uncomplicated: 17.3% \| 17.6%  complicated: 22.3% \| 21.4%  hypothyroidism: 14.9% \| 14.2%  obesity: 21.4% \| 23.3%  weight loss: 5.5% \| 5.6%  fluid and electrolyte disorders:  33.4% \| 30.6%  deficiency anaemia: 6.7% \| 5.5% | diabetes melitus: 15.1% \|16.2% |
| Mental & behavioural disorders | nicotine abuse: 18.5% \| 20.3%  psychosocial disorders: 6.1% \| 5.7% | depression: 5.2% \| 5.0% | dementia: 1.3% \| 1.5% |
| Nervous system |  |  | hemiplegia or paraplegi: 0.7% \| 0.8% |
| Circulatory system | arterial hypertension: 54.4% \| 61.4%  condition after cerebral insult:  3.4% \| 4.0% | cardiac arrhythmias: 64.2% \| 63.0%  valvular disease: 38.5% \| 39.9%  pulmonary circulation disorders:  17.3% \| 18.9%  peripheral vascular disorders:  11.5% \| 11.9%  hypertension  uncomplicated: 30.3% \| 28.4%  complicated: 48.1% \| 49.5% | myocardial infarction: 4.3% \| 4.2%  congestive heart failure: 29.8% \| 28.3%  peripheral vascular disease: 5.0% \| 5.4%  cerebrovascular disease: 2.2% \| 2.2% |
| Respiratory system | chronic obstructive pulmonary disease: 6.9% \| 8.1% | chronic pulmonary disease:  18.1% \| 18.9% | chronic pulmonary disease:  6.0% \| 6.7% |
| Digestive system |  |  | peptic ulcer disease: 0.2% \| 0.2%  liver disease  mild: 1.0% \| 0.9%  moderate or severe: 0.0% \| 0.1% |
| Musculoskeletal system | rheumatoid arthritis: 1.8% \| 1.6%  musculoskeletal diseases: 13.8% \| 11% |  | rheumatic disease: 1.1% \| 1.1% |
| Urogenital system | chronic renal insufficiency:  10.1% \| 10.5% | renal failure:  63.4% \| 62.5% | chronic kidney disease:  22.5% \| 23.0% |

^1^ = data for the entire study

Table S 11: Diagnosis stages: distribution of NYHA classes, CCI & CHA2DS2 VASc scores in 2020

| **6 König et al. (2022)** | | | **10 Ueberham et al. (2021)** | | | | | | |
| --- | --- | --- | --- | --- | --- | --- | --- | --- | --- |
| **NYHA** | | | **CCI** | | | **CHA_2_DS_2_-VASc-Score** | | | |
| Study  (n = 12.781)  NYHA I: N/A  NYHA II: 997 (7.7%)  NYHA III: 5.986 (46.4%)  NYHA IV: 5.798 (44.9%) | Control  (n = 13,526)  NYHA I: N/A  NYHA II: 1,265 (9.2%)  NYHA III: 6,200 (45.3%)  NYHA IV: 6,061(44.3%) | p-value  <0.001  <0.001  <0.001 | study (n = 7,001)  0-1: 4,823 (68.9%)  2-4: 1,855 (26.5%)  ≥ 5: 323 (4.6%) | control  (n = 8,424)  0-1: 5,728 (68%)  2-4: 2,280 (27.1%)  ≥ 5: 416 (4.9%) | p-value  0.23  0.43  0.35 | study (n = 7,001)  0-1: 1,605 (22.9%)  2-4: 4,118 (58.8%)  ≥ 5: 1,278 (18.3%) | control  (n = 8,424)  0-1: 1,868 (22.2%)  2-4: 5,191 (61.6%)  ≥ 5: 1,365 (16.2%) | p-value  0.27  <0.01  <0.01 |  |

N/A = not available

Table S 12: Number of & change in hospital admissions for cardiovascular diseases in 2020

|  |  |  | **Number of hospital admissions** | | | | **Number of hospital admissions per day** | | | | |  |
| --- | --- | --- | --- | --- | --- | --- | --- | --- | --- | --- | --- | --- |
| **No.** | **Publication** |  | **Study group** | **Control group** | **Relative change (95% CI)** | | **Study group** | **Control group** | | **IRR (95% CI) or**  **relative change** | |  |
| 2 | Bollmann  et al. (2020) | I48 | I) 1,739  II) 1,322 | I) N/A  II) 1,640 | II) -19.39%* | | I) 29.5  II) 21.7 | I) N/A  II) 26.9 | | I/20 zu II/19) 0.81  (0.75; 0.87). p<0.01  II) 0.74 (0.68; 0.79). p<0.01 | |  |
| 3 | Bollmann et al. (2021) | I50 | 14,197  I) N/A  II) 5,187  III) 9,101 | 15,206  I) N/A  II) 6,152  III) 9,054 | -6.64%*  II) -15.69%*  III) +0.52%* | | I) N/A  II) 55.2  III) 58.0 | I) N/A  II) 65.4  III) 57.7 | | I) N/A  II) 0.84 (0.81; 0.87). p<0.01  III) 1.01 (0.98; 1.03). p<0.01 | |  |
| 4 | Jaehn et al. (2021) | I50 | 1,938  I) 1,050  II) 888 | 2,032  I) 978  II) 1,054 | -4.63%*  I) +7.36%*  II) -15.75%* | |  |  | |  | |  |
| 7 | Slagman  et al. (2022) | I50 | 13,164  4 weeks median:  35 (IQR: 23-64) | 13,480  4 weeks median:  38 (IQR 26; 55) | median 2019/2020:  -1.74%  (-9.6%; 6.0%) | |  |  | |  | |  |
| 8 | Stöhr et al. (2020) | I50 | 489 | N/A | II) -38%. p=0.002 | |  |  | |  | |  |
| 9 | Stöwhas. Lippert (2021) | I48 | 50*  Self-presentation: 19  I) 9. II) 10  Contract doctor: 31  I) 7. II) 24 | 113*  Self-presentation: 36  Contract doctor: 77 | -55.75%*  Self-presentation:  -47.22%*  Contract doctor:  -59.74%* | | 0.36*  I) 0.31* II) 0.40*  Self-presentation:  I) 0.17. II) 0.12  Contract doctor: I) 0.13. II) 0.28 | 0.44*  Self-presentation:  0.14  Contract doctor: 0.30 | | -18.18%*  I) -29.55%* II) -9.09*  Self-presentation: 0.00%*  I) 24%. II) -17%  Contract doctor: -26.67%* I) -55%. II) -7% | |  |
|  |  | I50 | 85*  Self-presentation: 19  I) 7. II) 12  Contract doctor: 66  I) 25. II) 41 | 191*  Self-presentation: 51  Contract doctor: 140 | -55.50%*  Self-presentation:  -62.75%*  Contract doctor:  -52.86%* | | 0.62*  I) 0.61* II) 0.62*  Self-presentation:  I) 0.13. II) 0.14  Contract doctor: I) 0.48. II) 0.48 | 0.74*  Self-presentation: 0.20  Contract doctor: 0.54 | | -16.22%*  I) -17.57%* II) -16.22%*  Self-presentation: -30.00%* I) -32%. II) -30%  Contract doctor: -12% I) -12%. II) -12% | |  |
| 10 | Ueberham  et al. (2021) | I48 | 7,001  I) 1,331  II) 5,670  incident: 4,060 (58%)  prevalent: 2,941 (42%) | 8,424  I) 2,326  II) 6,098  incident: 5,004 (59.4%)  prevalent: 3,420(40.6%) | | -16.65%*  (-18%; -15%). p<0.001  I) -41.92%  II) -7.02%  incident: -14%  (-16%; -12%). p<0.001  prevalent: -19%  (-21%; -17%). p<0.001 | | |  | |  | |
|  |  |  | ER: 2,981 (51.2%)  regular: 2,843 (48.8%) | ER: 3,390 (48.3%)  regular: 3,626 (51.7%) | ER: -12.06%  regular: -21.59% | | |  | |  | |  |

* = calculated for the review, N/A = not available, IRR = Incidence Rate Ratio, ER = Emergency room, I/ II/ III = data set is divided into study-specific time periods

Table S 13: Number of diagnostics and treatment for I48 atrial fibrillation/flutter

|  | **Period** | **Study group** | **Control group** | **OR (95%-CI)** | **p-value** |
| --- | --- | --- | --- | --- | --- |
| **TEE** | I | 462 (34.7%) | 964 (41.4%) | 0.74 (0.64; 0.86) | < 0.01 |
|  | II | 2,262 (39.9%) | 2,450 (40.2%) | 0.96 (0.88; 1.03) | 0.26 |
| **ECV** | I | 540 (40.6%) | 924 (39.7%) | 1.08 (0.94; 1.25) | 0.27 |
|  | II | 2,188 (38.6%) | 2,286 (37.5%) | 1.06 (0.98; 1.14) | 0.17 |
| **CA** | I | 288 (21.6%) | 491 (21.1%) | 0.98 (0.82; 1.17) | 0.82 |
|  | II | 1,299 (22.9%) | 1,309 (21.5%) | 1.05 (0.96; 1.16) | 0.28 |

TEE = transesophageal echocardiographies, ECV = electrical cardioversions, CA = catheter ablations, OR= Odds ratio, CI=confidence interval

Table S 14: Number of S/V prescriptions 2018 - 2023 (Kerwagen et al., 2023, p. 4f.)

| **Number of prescriptions**  **Study period** | **Relative change per quarter*** | **Number of prescriptions**  **Control period** | **Relative change per quarter*** |
| --- | --- | --- | --- |
| **prevalent** |  |  |  |
| *S/V total (S/V \| S/V + SGLT2i)*  **2020**  N = 720,364  Q1: 165,676 (154,620 (93.3%) \| 11,056 (6.7%))  Q2: 174,480 (161,763 (92.7%) \| 12,717 (7.3%))  Q3: 184,947 (170,448 (92.2%) \| 14,500 (7.8%))  Q4: 195,261 (177,760 (91.0%) \| 17,501 (9.0%))  **2021**  N = 914,480  Q1: 207,290 (184.194 (88.9%) \| 23,096 (11.1%))  Q2: 219,762 (188.633 (85.8%) \| 31,128 (14.2%))  Q3: 235,367 (192.310 (81.7%) \| 43,057 (18.3%))  Q4: 252,061 (186.971 (74.2%) \| 65,090 (25.8%))  **2022**  N = 1,176,368  Q1: 270,326 (182,817 (67.6%) \| 87,509 (32.4%))  Q2: 286,160 (175,685 (61.4%) \| 110,476 (38.6%))  Q3: 301,287 (170,785 (56.7%) \| 130,502 (43.3%))  Q4: 318,595 (166,561 (52.3%) \| 152,035 (47.7%))  **2023**  N = 688,725  Q1: 337,463 (163,538 (48.5%) \| 173,925 (51.5%))  Q2: 351,262 (158,833 (45.2%) \| 192,429 (54.8%)) | *S/V \| S/V + SGLT2i*  **2020**  Q4-Q1: -0.85% \| +13.56%  Q1-Q2: -0.64% \| +8.96%  Q2-Q3: -0.54% \| +6.85%  Q3-Q4: -1.30% \| +15.38%  **2021**  Q4-Q1: -2.31% \| +23.33%  Q1-Q2: -3.49% \| +27.93%  Q2-Q3: -4.78% \| +28.87%  Q3-Q4: -9.18% \| +40.98%  **2022**  Q4-Q1: -8.89% \| +25.58%  Q1-Q2: -9.17% \| +19.14%  Q2-Q3: -7.65% \| +12.18%  Q3-Q4: -7.76% \| +10.16%  **2023**  Q4-Q1: -7.27% \| +7.97%  Q1-Q2: -6.80% \| +6.41% | *S/V total (S/V \| S/V + SGLT2i)*  **2018**  N = 310,778  Q1: 63,632 (61,586 (96.8%) \| 2047 (3.2%))  Q2: 72,704 (70,129 (96.5%) \| 2576 (3.5%))  Q3: 81,644 (78,501 (96.2%) \| 3143 (3.8%))  Q4: 92,798 (88,926 (95.8%) \| 3872 (4.2%))  **2019**  N = 511,362  Q1: 107,069 (102,343 (95.6%) \| 4726 (4.4%))  Q2: 121,044 (115,240 (95.2%) \| 5804 (4.8%))  Q3: 134,080 (127,190 (94.9%) \| 6890 (5.1%))  Q4: 149,169 (140,347 (94.1%) \| 8822 (5.9%)) | *S/V \| S/V + SGLT2i*  **2018**  Q4-Q1: -0.41% \| +14.3%  Q1-Q2: -0.31% \| +9.38%  Q2-Q3: -0.31% \| +8.57%  Q3-Q4: -0.42% \| +10.53%  **2019**  Q4-Q1: -0.21% \| +4.76%  Q1-Q2: -0.42% \| +9.09%  Q2-Q3: -0.32% \| +6.25%  Q3-Q4: -0 84% \|+15.59% |
| **Incident** |  |  |  |
| *S/V total*  Q1/2020: 26,855 \| Q2/2020: 22,145  Q1/2021: 27,197  Q2/2023: 53,534 | N/A | N/A | N/A |

* calculated for the review, S/V = Sacubitril/Valsartan, SGLT2i = SGLT2 inhibitor, N/A = not available

Table S 15: Measures in cardiac rehabilitation in 2020 (Bestehorn et al., 2022, p. 25)

| **Rehabilitation measure** | **Measure per person** | | **Relative change**  **per person [%]** | | **Relative reduction**  **total* [%]** |
| --- | --- | --- | --- | --- | --- |
|  | **Study group** | **Control group** |  |  |  |
| Ergometer | 7.73 | 8.05 | | -3.97 | 20.9 |
| Terrain training | 4.49 | 5.12 | | -3.56 | 21.2 |
| Medical training therapy | 4.64 | 4.77 | | -2.77 | 20.2 |
| Gymnastics | 8.52 | 8.45 | | +0.81 | 17.7 |
| Physiotherapy | 4.78 | 4.42 | | +8.2 | 11.1 |
| Occupational therapy | 0.73 | 0.70 | | +5.2 | 13.2 |
| Psychological group training | 0.84 | 0.82 | | +2.46 | 16.2 |
| Psychological counselling session | 0.44 | 0.51 | | -14.39 | 29.6 |
| Relaxation | 2.19 | 2.34 | | -6.40 | 23.1 |
| Nutritional counselling | 1.0 | 0.97 | | +3.3 | 15.0 |
| Training kitchen | 0.3 | 0.44 | | -32.5 | 44.6 |
| Diabetes counselling | 0.39 | 0.44 | | -11.8 | 27.5 |
| Social counselling | 0.97 | 0.93 | | +5.0 | 13.5 |
| Doctor's rounds | 3.52 | 5.45 | | -35.4 | 44.6 |
| Nursing rounds/  wound management | 1.85 | 1.80 | | +2.3 | 15.5 |
| Lectures/ seminars | 4.82 | 5.86 | | -17.7 | 32.3 |
| Heart failure training | 0.3 | 0.36 | | -16.9 | 32.8 |

Table S 16: Cancer search string

| **PubMed 29.01.2024** |  |
| --- | --- |
| (((((((((((cancer*[Title/Abstract]) OR (neoplasia*[Title/Abstract])) OR (tumor*[Title/Abstract])) OR (malignant*[Title/Abstract])) OR (malignancy*[Title/Abstract])) OR (oncological*[Title/Abstract])) OR (oncology*[Title/Abstract])) OR (carcinoma*[Title/Abstract])) OR (neoplasms[MeSH Terms])) OR ((((((((((((((((((((((((((((((((((((((((((((((((((((((lung cancer*[Title/Abstract]) OR (lung carcinoma*[Title/Abstract])) OR (bronchial carcinoma*[Title/Abstract])) OR (lung neoplasms[MeSH Terms])) OR (bronchogenic carcinoma[MeSH Terms])) OR (pancreatic cancer*[Title/Abstract])) OR (pancreas cancer*[Title/Abstract])) OR (pancreatic carcinoma*[Title/Abstract])) OR (pancreas carcinoma*[Title/Abstract])) OR (pancreatic neoplasms[MeSH Terms])) OR (Breast cancer*[Title/Abstract])) OR (mammary cancer*[Title/Abstract])) OR (breast carcinoma*[Title/Abstract])) OR (mammary carcinoma*[Title/Abstract])) OR (breast neoplasms[MeSH Terms])) OR (prostate cancer*[Title/Abstract])) OR (prostate carcinoma*[Title/Abstract])) OR (prostatic neoplasms[MeSH Terms])) OR (colon cancer*[Title/Abstract])) OR (colon carcinoma*[Title/Abstract])) OR (colorectal cancer*[Title/Abstract])) OR (colorectal carcinoma*[Title/Abstract])) OR (bowel cancer*[Title/Abstract])) OR (rectal cancer*[Title/Abstract])) OR (rectal carcinoma*[Title/Abstract])) OR (colonic neoplasms[MeSH Terms])) OR (colorectal neoplasms[MeSH Terms])) OR (rectal neoplasms[MeSH Terms])) OR (liver cancer*[Title/Abstract])) OR (liver carcinoma*[Title/Abstract])) OR (hepatic cancer*[Title/Abstract])) OR (hepatic carcinoma*[Title/Abstract])) OR (liver neoplasms[MeSH Terms])) OR (stomach cancer*[Title/Abstract])) OR (gastric cancer*[Title/Abstract])) OR (stomach carcinoma*[Title/Abstract])) OR (gastric carcinoma*[Title/Abstract])) OR (stomach neoplasms[MeSH Terms])) OR (brain tumor*[Title/Abstract])) OR (cerebral tumor*[Title/Abstract])) OR (brain neoplasms[MeSH Terms])) OR (melanocarcinoma*[Title/Abstract])) OR (malignant melanoma*[Title/Abstract])) OR (melanoma[MeSH Terms])) OR (non-hodgkin's lymphoma*[Title/Abstract])) OR (non-hodgkin-lymphoma[MeSH Terms])) OR (bladder cancer*[Title/Abstract])) OR (bladder carcinoma*[Title/Abstract])) OR (urinary bladder neoplasms[MeSH Terms])) OR (kidney cancer*[Title/Abstract])) OR (kidney carcinoma*[Title/Abstract])) OR (renal cancer*[Title/Abstract])) OR (renal carcinoma*[Title/Abstract])) OR (kidney neoplasms[MeSH Terms]))) AND (((((sars-cov-2) OR (2019-ncov)) OR (pandemic)) OR (coronavirus)) OR (covid-19))) AND ((german) OR (germany))) | 1,302 |

| **Web of Science 02.02.2024** |  |
| --- | --- |
| ((TS=(((((((((((cancer*) OR (neoplasia*)) OR (tumor*)) OR (malignant*)) OR (malignancy*)) OR (oncological*)) OR (oncology*)) OR (carcinoma*)) OR (neoplasms)) OR (((((((((((((((((((((((((((((((((((((((((((((((((((((("lung cancer*") OR ("lung carcinoma*")) OR ("bronchial carcinoma*")) OR ("lung neoplasms")) OR ("bronchogenic carcinoma")) OR ("pancreatic cancer*")) OR ("pancreas cancer*")) OR ("pancreatic carcinoma*")) OR ("pancreas carcinoma*")) OR ("pancreatic neoplasms")) OR ("Breast cancer*")) OR ("mammary cancer*")) OR ("breast carcinoma*")) OR ("mammary carcinoma*")) OR ("breast neoplasms")) OR ("prostate cancer*")) OR ("prostate carcinoma*")) OR ("prostatic neoplasms")) OR ("colon cancer*")) OR ("colon carcinoma*")) OR ("colorectal cancer*")) OR ("colorectal carcinoma*")) OR ("bowel cancer*")) OR ("rectal cancer*")) OR ("rectal carcinoma*")) OR ("colonic neoplasms")) OR ("colorectal neoplasms")) OR ("rectal neoplasms")) OR ("liver cancer*")) OR ("liver carcinoma*")) OR ("hepatic cancer*")) OR ("hepatic carcinoma*")) OR ("liver neoplasms")) OR ("stomach cancer*")) OR ("gastric cancer*")) OR ("stomach carcinoma*")) OR ("gastric carcinoma*")) OR ("stomach neoplasms")) OR ("brain tumor*")) OR ("cerebral tumor*")) OR ("brain neoplasms")) OR (melanocarcinoma*)) OR ("malignant melanoma*")) OR (melanoma)) OR ("non-hodgkin's lymphoma*")) OR (non-hodgkin-lymphoma)) OR ("bladder cancer*")) OR ("bladder carcinoma*")) OR ("urinary bladder neoplasms")) OR ("kidney cancer*")) OR ("kidney carcinoma*")) OR ("renal cancer*")) OR ("renal carcinoma*")) OR ("kidney neoplasms"))) )) AND ALL=((((((((sars-cov-2 ) OR (2019-ncov )) OR (pandemic )) OR (coronavirus )) OR (covid-19 )))) )) AND ALL=(((german) OR (Germany))) | 1,438 |

| **Cochrane Library 06.02.2024** |  |
| --- | --- |
| ((cancer*:ti,ab) OR (neoplasia*:ti,ab) OR (tumor*:ti,ab) OR (malignant*:ti,ab) OR (malignancy*:ti,ab) OR (oncological*:ti,ab) OR (oncology*:ti,ab) OR (carcinoma*:ti,ab) OR (neoplasms*:ti,ab) OR ((lung cancer*):ti,ab) OR ((lung carcinoma*):ti,ab) OR ((bronchial carcinoma*):ti,ab) OR ((lung neoplasms*):ti,ab) OR ((bronchogenic carcinoma*):ti,ab) OR ((pancreatic cancer*):ti,ab) OR ((pancreas cancer*):ti,ab) OR ((pancreatic carcinoma*):ti,ab) OR ((pancreas carcinoma*):ti,ab) OR ((pancreatic neoplasms*):ti,ab) OR ((Breast cancer*):ti,ab) OR ((mammary cancer*):ti,ab) OR ((breast carcinoma*):ti,ab) OR ((mammary carcinoma*):ti,ab) OR ((breast neoplasms*):ti,ab) OR ((prostate cancer*):ti,ab) OR ((prostate carcinoma*):ti,ab) OR ((prostatic neoplasms*):ti,ab) OR ((colon cancer*):ti,ab) OR ((colon carcinoma*):ti,ab) OR ((colorectal cancer*):ti,ab) OR ((colorectal carcinoma*):ti,ab) OR ((bowel cancer*):ti,ab) OR ((rectal cancer*):ti,ab) OR ((rectal carcinoma*):ti,ab) OR ((colonic neoplasms*):ti,ab) OR ((colorectal neoplasms*):ti,ab) OR ((rectal neoplasms*):ti,ab) OR ((liver cancer*):ti,ab) OR ((liver carcinoma*):ti,ab) OR ((hepatic cancer*):ti,ab) OR ((hepatic carcinoma*):ti,ab) OR ((liver neoplasms*):ti,ab) OR ((stomach cancer*):ti,ab) OR ((gastric cancer*):ti,ab) OR ((stomach carcinoma*):ti,ab) OR ((gastric carcinoma*):ti,ab) OR ((stomach neoplasms*):ti,ab) OR ((brain tumor*):ti,ab) OR ((cerebral tumor*):ti,ab) OR ((brain neoplasms*):ti,ab) OR ((melanocarcinoma*):ti,ab) OR ((malignant melanoma*):ti,ab) OR (melanoma*:ti,ab) OR ((non hodgkin's lymphoma*):ti,ab) OR ((non Hodgkin lymphoma*):ti,ab) OR ((bladder cancer*):ti,ab) OR ((bladder carcinoma*):ti,ab) OR ((urinary bladder neoplasms*):ti,ab) OR ((kidney cancer*):ti,ab) OR ((kidney carcinoma*):ti,ab) OR ((renal cancer*):ti,ab) OR ((renal carcinoma*):ti,ab) OR ((kidney neoplasms*):ti,ab)) AND ((sars cov 2) OR (2019 ncov) OR (pandemic) OR (coronavirus) OR (Covid 19)) AND ((german) OR (Germany)) | 30 trails |

| **Scopus 29.02.2024** |  |
| --- | --- |
| ( ( TITLE-ABS ( cancer* ) OR TITLE-ABS ( neoplasia* ) OR TITLE-ABS ( tumor* ) OR TITLE-ABS ( malignant* ) OR TITLE-ABS ( malignancy* ) OR TITLE-ABS ( oncological* ) OR TITLE-ABS ( oncology* ) OR TITLE-ABS ( carcinoma* ) OR TITLE-ABS ( neoplasms* ) OR TITLE-ABS ( "lung cancer*" ) OR TITLE-ABS ( "lung carcinoma*" ) OR TITLE-ABS ( "bronchial carcinoma*" ) OR TITLE-ABS ( "lung neoplasms*" ) OR TITLE-ABS ( "bronchogenic carcinoma*" ) OR TITLE-ABS ( "pancreatic cancer*" ) OR TITLE-ABS ( "pancreas cancer*" ) OR TITLE-ABS ( "pancreatic carcinoma*" ) OR TITLE-ABS ( "pancreas carcinoma*" ) OR TITLE-ABS ( "pancreatic neoplasms*" ) OR TITLE-ABS ( "Breast cancer*" ) OR TITLE-ABS ( "mammary cancer*" ) OR TITLE-ABS ( "breast carcinoma*" ) OR TITLE-ABS ( "mammary carcinoma*" ) OR TITLE-ABS ( "breast neoplasms*" ) OR TITLE-ABS ( "prostate cancer*" ) OR TITLE-ABS ( "prostate carcinoma*" ) OR TITLE-ABS ( "prostatic neoplasms*" ) OR TITLE-ABS ( "colon cancer*" ) OR TITLE-ABS ( "colon carcinoma*" ) OR TITLE-ABS ( "colorectal cancer*" ) OR TITLE-ABS ( "colorectal carcinoma*" ) OR TITLE-ABS ( "bowel cancer*" ) OR TITLE-ABS ( "rectal cancer*" ) OR TITLE-ABS ( "rectal carcinoma*" ) OR TITLE-ABS ( "colonic neoplasms*" ) OR TITLE-ABS ( "colorectal neoplasms*" ) OR TITLE-ABS ( "rectal neoplasms*" ) OR TITLE-ABS ( "liver cancer*" ) OR TITLE-ABS ( "liver carcinoma*" ) OR TITLE-ABS ( "hepatic cancer*" ) OR TITLE-ABS ( "hepatic carcinoma*" ) OR TITLE-ABS ( "liver neoplasms*" ) OR TITLE-ABS ( "stomach cancer*" ) OR TITLE-ABS ( "gastric cancer*" ) OR TITLE-ABS ( "stomach carcinoma*" ) OR TITLE-ABS ( "gastric carcinoma*" ) OR TITLE-ABS ( "stomach neoplasms*" ) OR TITLE-ABS ( "brain tumor*" ) OR TITLE-ABS ( "cerebral tumor*" ) OR TITLE-ABS ( "brain neoplasms*" ) OR TITLE-ABS ( melanocarcinoma* ) OR TITLE-ABS ( "malignant melanoma*" ) OR TITLE-ABS ( melanoma* ) OR TITLE-ABS ( "non-hodgkin's lymphoma*" ) OR TITLE-ABS ( non-hodgkin-lymphoma* ) OR TITLE-ABS ( "bladder cancer*" ) OR TITLE-ABS ( "bladder carcinoma*" ) OR TITLE-ABS ( "urinary bladder neoplasms*" ) OR TITLE-ABS ( "kidney cancer*" ) OR TITLE-ABS ( "kidney carcinoma*" ) OR TITLE-ABS ( "renal cancer*" ) OR TITLE-ABS ( "renal carcinoma*" ) OR TITLE-ABS ( "kidney neoplasms*" ) ) AND ( TITLE-ABS-KEY ( ( sars-cov-2 ) OR ( 2019-ncov ) OR ( pandemic ) OR ( coronavirus ) OR ( covid-19 ) ) ) AND ( TITLE-ABS-KEY ( ( german ) OR ( germany ) ) ) ) | 321 |

| **Embase 29.01.2024** |  |
| --- | --- |
| ((cancer*:ti,ab OR neoplasia*:ti,ab OR tumor*:ti,ab OR malignant*:ti,ab OR malignancy*:ti,ab OR oncological*:ti,ab OR oncology*:ti,ab OR carcinoma*:ti,ab OR neoplasms*:ti,ab)  OR ('lung cancer*':ti,ab OR 'lung carcinoma*':ti,ab OR 'bronchial carcinoma*':ti,ab OR 'lung neoplasms*’:ti,ab  OR 'bronchogenic carcinoma*’:ti,ab   OR 'pancreatic cancer*':ti,ab OR 'pancreas cancer*':ti,ab OR 'pancreatic carcinoma*':ti,ab OR 'pancreas carcinoma*':ti,ab OR 'pancreatic neoplasms*’:ti,ab   OR 'Breast cancer*':ti,ab OR 'mammary cancer*':ti,ab OR 'breast carcinoma*':ti,ab OR 'mammary carcinoma*':ti,ab OR 'breast neoplasms*’:ti,ab   OR 'prostate cancer*':ti,ab OR 'prostate carcinoma*':ti,ab OR 'prostatic neoplasms*’:ti,ab   OR 'colon cancer*':ti,ab OR 'colon carcinoma*':ti,ab OR 'colorectal cancer*':ti,ab OR 'colorectal carcinoma*':ti,ab OR 'bowel cancer*':ti,ab OR 'rectal cancer*':ti,ab OR 'rectal carcinoma*':ti,ab OR 'colonic neoplasms*’:ti,ab   OR 'colorectal neoplasms*’:ti,ab   OR 'rectal neoplasms*’:ti,ab   OR 'liver cancer*':ti,ab OR 'liver carcinoma*':ti,ab OR 'hepatic cancer*':ti,ab OR 'hepatic carcinoma*':ti,ab OR 'liver neoplasms*’:ti,ab   OR 'stomach cancer*':ti,ab OR 'gastric cancer*':ti,ab OR 'stomach carcinoma*':ti,ab OR 'gastric carcinoma*':ti,ab OR 'stomach neoplasms*’:ti,ab   OR 'brain tumor*':ti,ab OR 'cerebral tumor*':ti,ab OR 'brain neoplasms*’:ti,ab   OR melanocarcinoma*:ti,ab OR 'malignant melanoma*':ti,ab OR melanoma*:ti,ab OR 'non-hodgkin's lymphoma*':ti,ab OR non-hodgkin-lymphoma*:ti,ab   OR 'bladder cancer*':ti,ab OR 'bladder carcinoma*':ti,ab OR 'urinary bladder neoplasms*’:ti,ab   OR 'kidney cancer*':ti,ab OR 'kidney carcinoma*':ti,ab OR 'renal cancer*':ti,ab OR 'renal carcinoma*':ti,ab OR 'kidney neoplasms*’:ti,ab)) AND (sars-cov-2:ti,ab,kw OR 2019-ncov:ti,ab,kw OR pandemic:ti,ab,kw OR coronavirus:ti,ab,kw OR covid-19:ti,ab,kw) AND (german:ti,ab,kw OR germany:ti,ab,kw) | 390 |

Table S 17: CVD search string

| **PubMed 16.05.2024 & 27.05.2024** |  |
| --- | --- |
| **#CVD OR #I25 OR #I21 OR #I50 OR #I11 OR #I48 OR #I63 OR #I69 OR #I13 OR #I35 OR #I64**  "cardiovascular diseases"[MeSH Terms] OR "heart diseases"[MeSH Terms] OR "vascular diseases"[MeSH Terms] OR "cardiovascular disease*"[Title/Abstract] OR "cardiovascular disorder*"[Title/Abstract] OR "disease of the circulatory system"[Title/Abstract] OR "CVD"[Title/Abstract] OR "heart disease*"[Title/Abstract] OR "vascular disease*"[Title/Abstract] OR "kardiovaskulare erkrankung*"[Title/Abstract] OR "Myocardial Ischemia"[MeSH Terms] OR "ischamische herzkrankheit*"[Title/Abstract] OR "koronare herzkrankheit*"[Title/Abstract] OR "KHK"[Title/Abstract] OR "chronic ischemic heart disease*"[Title/Abstract] OR "coronary heart disease*"[Title/Abstract] OR "atherosclerotic cardiovascular disease*"[Title/Abstract] OR "atherosclerotic heart disease*"[Title/Abstract] OR "old myocardial infarct*"[Title/Abstract] OR "aneurysm of heart"[Title/Abstract] OR "ischaemic cardiomyopathy"[Title/Abstract] OR "myocardial ischaemia"[Title/Abstract] OR "Myocardial Infarction"[MeSH Terms] OR "ST Elevation Myocardial Infarction"[MeSH Terms] OR "Myokardinfarkt"[Title/Abstract] OR "Herzinfarkt"[Title/Abstract] OR "STEMI"[Title/Abstract] OR "NSTEMI"[Title/Abstract] OR "myocardial infarct*"[Title/Abstract] OR "cardial infarct*"[Title/Abstract] OR "heart attack*"[Title/Abstract] OR "heart failure"[MeSH Terms] OR "Herzinsuffizienz"[Title/Abstract] OR "heart insufficienc*"[Title/Abstract] OR "heart failure"[Title/Abstract] OR "left ventricular failure*"[Title/Abstract] OR "hypertensive heart disease*"[Title/Abstract] OR "Atrial Fibrillation"[MeSH Terms] OR "atrial flutter"[MeSH Terms] OR "Vorhofflimmern"[Title/Abstract] OR "atrial flutter"[Title/Abstract] OR "Atrial Fibrillation"[Title/Abstract] OR "Cerebral Infarction"[MeSH Terms] OR "Brain Infarction"[MeSH Terms] OR "stroke"[MeSH Terms] OR "Hirninfarkt"[Title/Abstract] OR "Schlaganfall"[Title/Abstract] OR "Apoplex"[Title/Abstract] OR "cerebral infarct*"[Title/Abstract] OR "cerebellar infarct*"[Title/Abstract] OR "brain infarct*"[Title/Abstract] OR "cerebrovascular infarct*"[Title/Abstract] OR "stroke"[Title/Abstract] OR "apoplexy"[Title/Abstract] OR "cerebrovascular accident"[Title/Abstract] OR "CVA"[Title/Abstract] OR "vascular accident"[Title/Abstract] OR "Cerebrovascular Disorders"[MeSH Terms] OR "sequelae of cerebrovascular disease*"[Title/Abstract] OR "cerebrovascular disease*"[Title/Abstract] OR "hypertensive heart and renal disease*"[Title/Abstract] OR "hypertensive heart disease*"[Title/Abstract] OR "hypertensive kidney disease*"[Title/Abstract] OR "hypertensive renal disease*"[Title/Abstract] OR "Aortic Valve Disease"[MeSH Terms] OR "Aortenklappenstenose"[Title/Abstract] OR "nonrheumatic aortic valve disorder*"[Title/Abstract] OR "Aortic valve stenosis"[Title/Abstract] OR "Aortic stenosis"[Title/Abstract] OR "aortic valve insufficienc*"[Title/Abstract] OR "aortic insufficienc*"[Title/Abstract] OR "aortic valve disorder*"[Title/Abstract] OR "stroke not specified as haemorrhage or infarct*"[Title/Abstract] | 16.05.:  3,156,641  27.05.:  3,160,418 |
| **AND Corona**  "Pandemics"[MeSH Terms] OR "pandemic*"[All Fields] OR "Pandemie"[All Fields] OR "COVID-19"[MeSH Terms] OR "covid*"[All Fields] OR "sars-cov-2"[MeSH Terms] OR "sars*"[All Fields] OR "coronavirus"[MeSH Terms] OR "coronavirus"[MeSH Terms] OR "coronavirus"[All Fields] OR "coronaviruses"[All Fields] OR "corona"[All Fields] OR "coronae"[All Fields] OR "coronas"[All Fields] OR "2019-nCov"[All Fields] OR "CoV"[All Fields] | 16.05.:  28,896  27.05.:  28,991 |
| **AND Germany**  "germany"[MeSH Terms] OR "german*"[All Fields] OR "deutsch*"[All Fields] | 16.05.: 2,054  27.05.: 2,061 |
| **Filter: from 2018 – 2024**  AND (2018:2024[pdat]) | 16.05.: 1,935  **27.05.: 1,942** |

| **Web of Science 16.05. & 27.05.2024** |  |
| --- | --- |
| **#CVD OR #I25 OR #I21 OR #I50 OR #I11 OR #I48 OR #I63 OR #I69 OR #I13 OR #I35 OR #I64**  TS=("cardiovascular disease*" OR "heart disease*" OR "vascular disease*" OR "cardiovascular disorder*" OR "disease* of the circulatory system" OR "CVD" OR "heart disease*" OR "vascular disease*" OR "kardiovaskulare erkrankung*" OR "Myocardial Ischemia" OR "ischamische herzkrankheit*" OR "koronare herzkrankheit*" OR "KHK" OR "chronic ischemic heart disease*" OR "coronary heart disease*" OR "atherosclerotic cardiovascular disease*" OR "atherosclerotic heart disease*" OR "old myocardial infarct*" OR "aneurysm of heart" OR "ischaemic cardiomyopathy" OR "myocardial ischaemia" OR "Myocardial Infarction" OR "ST Elevation Myocardial Infarction" OR "Myokardinfarkt" OR "Herzinfarkt" OR "STEMI" OR "NSTEMI" OR "myocardial infarct*" OR "cardial infarct*" OR "heart attack*" OR "heart failure" OR "Herzinsuffizienz" OR "heart insufficienc*" OR "heart failure" OR "left ventricular failure*" OR "hypertensive heart disease*" OR "Atrial Fibrillation" OR "atrial flutter" OR "Vorhofflimmern" OR "atrial flutter" OR "Atrial Fibrillation" OR "Cerebral Infarction" OR "Brain Infarction" OR "stroke" OR "Hirninfarkt" OR "Schlaganfall" OR "Apoplex" OR "cerebral infarct*" OR "cerebellar infarct*" OR "brain infarct*" OR "cerebrovascular infarct*" OR "stroke" OR "apoplexy" OR "cerebrovascular accident" OR "CVA" OR "vascular accident" OR "Cerebrovascular Disorders" OR "sequelae of cerebrovascular disease*" OR "cerebrovascular disease*" OR "hypertensive heart and renal disease*" OR "hypertensive heart disease*" OR "hypertensive kidney disease*" OR "hypertensive renal disease*" OR "Aortic Valve Disease" OR "Aortenklappenstenose" OR "nonrheumatic aortic valve disorder*" OR "Aortic valve stenosis" OR "Aortic stenosis" OR "aortic valve insufficienc*" OR "aortic insufficienc*" OR "aortic valve disorder*" OR "stroke not specified as haemorrhage or infarct*") |  |
| **AND Corona**  ALL=("pandemic*" OR "Pandemie" OR "COVID-19" OR "covid*" OR CoV OR "sars-cov-2" OR "sars*" OR "coronavirus*" OR corona OR "2019-nCov") |  |
| **AND Germany**  ALL=("german*" OR "deutsch*") |  |
| **Filter: from 2018 – 2024** | 16.05.: 1,267  **27.05.: 1,275** |

| **Cochrane Library 16.05. & 27.05.2024** | |  |
| --- | --- | --- |
| CVD | MeSH descriptor: Cardiovascular Diseases OR MeSH descriptor: Vascular Diseases OR MeSH descriptor: Heart Diseases OR  (("cardiovascular disease*" OR "heart disease*" OR "vascular disease*" OR “cardiovascular disorder*” OR “disease* of the circulatory system” OR CVD OR “Krankheit* des Kreislaufsystems” OR “Krankheit* des Herzkreislaufsystem*” OR “Herz-Kreislauf-Krankheit*” OR “kardiovaskuläre Erkrankung*“):ti,ab,kw) |  |
| I25 | MeSH descriptor: Coronary Disease OR  ((„ischämische Herzkrankheit*“ OR “atherosklerotische Herzkrankheit*“ OR „alter Myokardinfarkt“ OR „Herz-Aneurysma“ OR „Herz-Wand-Aneurysma“ OR „Koronararterienaneurysma“ OR „ischämische Kardiomyopathie“ OR „Myokardischämie“ OR „Koronare Herzkrankheit*“ OR KHK OR “chronic ischemic heart disease*” OR “coronary heart disease*” OR “atherosclerotic cardiovascular disease*“ OR “atherosclerotic heart disease*“ OR „old myocardial infarct*“ OR „aneurysm of heart“ OR „coronary artery aneurysm and dissection“ OR „ischaemic cardiomyopathy“ OR “myocardial ischaemia“):ti,ab,kw) |  |
| I21 | MeSH descriptor: Myocardial Infarction OR  Myokardinfarkt OR Herzinfarkt OR STEMI OR NSTEMI OR “myocardial infarct*” OR "ST Elevation Myocardial Infarction" OR “cardial infarct*” OR “heart attack*”):ti,ab,kw) |  |
| I50 | MeSH descriptor: Heart Failure OR  (("*heart failure*" OR Herzinsuffizienz OR “heart insufficiency”):ti,ab,kw) |  |
| I11 | (("Hypertensive Herzkrankheit*" OR "hypertensive heart disease*"):ti,ab,kw) |  |
| I48 | MeSH descriptor: Atrial Fibrillation OR MeSH descriptor: Atrial Flutter OR  (("Atrial Fibrillation" OR "Atrial Flutter" OR Vorhofflattern OR Vorhofflimmern):ti,ab,kw) |  |
| I63 | MeSH descriptor: Cerebral Infarction OR MeSH descriptor: Brain Infarction OR MeSH descriptor: Stroke OR  (Schlaganfall OR Apoplex OR Hirninfarkt OR "Zerebrovaskulärer Insult" OR "Ischämischer Insult“ OR "zerebral* Ischämie“ OR Hirninsult):ti,ab.kw) OR ("cerebral infarct*“ OR "cerebellar infarct*“ OR “brain infarct*“ OR "Cerebrovascular infarct*" OR stroke OR apoplexy OR "cerebrovascular accident" OR CVA OR "vascular accident"):ti,ab,kw) |  |
| I69 | MeSH descriptor: Cerebrovascular Disorders OR  ("Folgen einer zerebrovaskulären Kakheti*" OR "Zerebrovaskuläre Krankheit*" OR "Sequelae of cerebrovascular disease*" OR "Cerebrovascular disease*"):ti,ab,kw) |  |
| I13 | ("Hypertensive Herz- und Nierenkrankheit*" OR "Hypertensive Herzkrankheit*" OR "Hypertensive Nierenkrankheit*" OR "hypertensive heart and renal disease*" OR "hypertensive heart disease*" OR "hypertensive kidney disease*" OR "hypertensive renal disease*"):ti,ab,kw) |  |
| I35 | MeSH descriptor: Aortic Valve Disease OR MeSH descriptor: Aortic Valve Stenosis OR MeSH descriptor: Aortic Valve Insufficiency OR  ("Nichtrheumatische Aortenklappenkrankheit*" OR Aortenklappenstenose OR Aortenklappeninsuffizienz OR "Nonrheumatic aortic valve disorder*" OR "Aortic valve stenosis" OR "Aortic stenosis" OR "Aortic valve insufficienc*" OR "Aortic insufficienc*" OR "Aortic valve disorder*"):ti,ab,kw) |  |
| I64 | („Schlaganfall, nicht als Blutung oder Infarkt bezeichnet“):ti,ab,kw) |  |
|  | **#CVD OR #I25 OR #I21 OR #I50 OR #I11 OR #I48 OR #I63 OR #I69 OR #I13 OR #I35 OR #I64** | 1,272,899 |
|  | **AND Corona**  MeSH descriptor: Pandemics OR  (pandemic* OR Pandemie OR "COVID-19" OR covid* OR CoV OR "sars-cov-2" OR sars* OR coronavirus OR corona OR “2019 nCov“) | 17,844 |
|  | **AND Germany**  MeSH descriptor: Germany OR (german* OR deutsch*) | 901 |
|  | **Filter: from 2018 – 2024** | 823 (all types)  408 (Trail)  16.05.: 408  **27.05. 408** |

| **Scopus 24.05. & 27.05.2024** | |  |
| --- | --- | --- |
| CVD | TITLE-ABS ("cardiovascular disease*" OR "cardiovascular disease*" OR "cardiovascular disorder*" OR "disease* of the circulatory system" OR cvd OR "heart disease*" OR "vascular disease*" OR "Krankheit* des Kreislaufsystems" OR "Krankheit* des Herzkreislaufsystem*" OR "Herz-Kreislauf-Krankheit*" OR "kardiovaskuläre Erkrankung*“) | 620,407 |
| I25 | TITLE-ABS ("chromic ischemic heart disease*" OR "coronary heart disease*" OR "atherosclerotic cardiovascular disease*" OR "atherosclerotic heart disease*" OR "old myocardial infarct*" OR "aneurysm of heart" OR "coronary artery aneurysm and dissection" OR "ischaemic cardiomyopathy" OR "myocardial ischaemia" OR "ischämische Herzkrankheit*" OR "atherosklerotische Herz-Kreislauf-Krankheit*" OR "atherosklerotische Herzkrankheit*" OR "alter Myokardinfarkt" OR "Herz-Aneurysma" OR "Herz-Wand-Aneurysma" OR "Koronararterienaneurysma" OR "ischämische Kardiomyopathie" OR "Myokardischämie" OR "Koronare Herzkrankheit*" OR khk) | 129,843 |
| I21 | TITLE-ABS (myokardinfarkt OR herzinfarkt OR "ST-Hebungsinfarkt" OR stemi OR nstemi OR "myocardial infarct*" OR "cardial infarct*" OR "heart attack*" OR "ST Elevation Myocardial Infarction“) | 273,800 |
| I50 | TITLE-ABS ("Herzinsuffizienz" OR "heart insufficienc*" OR "*heart failure*" OR "left ventricular failure*“) | 255,810 |
| I11 | TITLE-ABS ("Hypertensive Herzkrankheit" OR "hypertensive heart disease*“) | 2,321 |
| I48 | TITLE-ABS (vorhofflattern OR vorhofflimmern OR "Atrial flutter" OR "atrial fibrillation“) | 112,627 |
| I63 | TITLE-ABS (Hirninfarkt OR "Zerebrovaskulärer Insult" OR Schlaganfall OR apoplex OR "Ischämischer Insult" OR "zerebral* Ischämie" OR "Hirninsult" OR "cerebral infarct*" OR "cerebellar infarct*" OR "brain infarct*" OR "Cerebrovascular infarct*" OR stroke OR apoplexy OR "cerebrovascular accident" OR cva OR "vascular accident“) | 478,444 |
| I69 | TITLE-ABS ("Folgen einer zerebrovaskulären Krankheit*" OR "Zerebrovaskuläre Krankheit*" OR "Sequelae of cerebrovascular disease*" OR "Cerebrovascular disease*" OR "Cerebrovascular Disorders“) | 35,068 |
| I13 | TITLE-ABS ("Hypertensive Herz- und Nierenkrankheit*" OR "Hypertensive Herzkrankheit*" OR "Hypertensive Nierenkrankheit*" OR "hypertensive heart and renal disease*" OR "hypertensive heart disease*" OR "hypertensive kidney disease*" OR "hypertensive renal disease*“) | 2,667 |
| I35 | TITLE-ABS („Nichtrheumatische Aortenklappenkrankheit*" OR aortenklappenstenose OR aortenklappeninsuffizienz OR "Nonrheumatic aortic valve disorder*" OR "Aortic valve stenosis" OR "Aortic stenosis" OR "Aortic valve insufficienc*" OR "Aortic insufficienc*" OR "Aortic valve disorder*" OR "Aortic Valve Disease“) | 34,616 |
| I64 | TITLE-ABS („Schlaganfall, nicht als Blutung oder Infarkt bezeichnet" OR "stroke, not specified as haemorrhage or infarct*“) | 7 |
|  | **#CVD OR #I25 OR #I21 OR #I50 OR #I11 OR #I48 OR #I63 OR #I69 OR #I13 OR #I35 OR #I64 (Title & Abstract)**  TITLE-ABS ("cardiovascular disease*" OR "cardiovascular disease*" OR "cardiovascular disorder*" OR "disease* of the circulatory system" OR cvd OR "heart disease*" OR "vascular disease*" OR "Krankheit* des Kreislaufsystems" OR "Krankheit* des Herzkreislaufsystem*" OR "Herz-Kreislauf-Krankheit*" OR "kardiovaskuläre Erkrankung*") OR TITLE-ABS ("chromic ischemic heart disease*" OR "coronary heart disease*" OR "atherosclerotic cardiovascular disease*" OR "atherosclerotic heart disease*" OR "old myocardial infarct*" OR "aneurysm of heart" OR "coronary artery aneurysm and dissection" OR "ischaemic cardiomyopathy" OR "myocardial ischaemia" OR "ischämische Herzkrankheit*" OR "atherosklerotische Herz-Kreislauf-Krankheit*" OR "atherosklerotische Herzkrankheit*" OR "alter Myokardinfarkt" OR "Herz-Aneurysma" OR "Herz-Wand-Aneurysma" OR "Koronararterienaneurysma" OR "ischämische Kardiomyopathie" OR "Myokardischämie" OR "Koronare Herzkrankheit*" OR khk) OR TITLE-ABS (myokardinfarkt OR herzinfarkt OR "ST-Hebungsinfarkt" OR stemi OR nstemi OR "myocardial infarct*" OR "cardial infarct*" OR "heart attack*" OR "ST Elevation Myocardial Infarction") OR TITLE-ABS ("Herzinsuffizienz" OR "heart insufficienc*" OR "*heart failure*" OR "left ventricular failure*") OR TITLE-ABS ("Hypertensive Herzkrankheit" OR "hypertensive heart disease*") OR TITLE-ABS (vorhofflattern OR vorhofflimmern OR "Atrial flutter" OR "atrial fibrillation") OR TITLE-ABS (hirninfarkt OR "Zerebrovaskulärer Insult" OR schlaganfall OR apoplex OR "Ischämischer Insult" OR "zerebral* Ischämie" OR "Hirninsult" OR "cerebral infarct*" OR "cerebellar infarct*" OR "brain infarct*" OR "Cerebrovascular infarct*" OR stroke OR apoplexy OR "cerebrovascular accident" OR cva OR "vascular accident") OR TITLE-ABS ("Folgen einer zerebrovaskulären Krankheit*" OR "Zerebrovaskuläre Krankheit*" OR "Sequelae of cerebrovascular disease*" OR "Cerebrovascular disease*" OR "Cerebrovascular Disorders") OR TITLE-ABS ("Hypertensive Herz- und Nierenkrankheit*" OR "Hypertensive Herzkrankheit*" OR "Hypertensive Nierenkrankheit*" OR "hypertensive heart and renal disease*" OR "hypertensive heart disease*" OR "hypertensive kidney disease*" OR "hypertensive renal disease*") OR TITLE-ABS ("Nichtrheumatische Aortenklappenkrankheit*" OR aortenklappenstenose OR aortenklappeninsuffizienz OR "Nonrheumatic aortic valve disorder*" OR "Aortic valve stenosis" OR "Aortic stenosis" OR "Aortic valve insufficienc*" OR "Aortic insufficienc*" OR "Aortic valve disorder*" OR "Aortic Valve Disease") OR TITLE-ABS ("Schlaganfall, nicht als Blutung oder Infarkt bezeichnet" OR "stroke, not specified as haemorrhage or infarct*") | 24.05:  1,563,579  27.05.:  1,564,494 |
|  | **AND Corona**  TITLE-ABS-KEY (pandemic* OR pandemie OR "COVID-19" OR covid* OR cov OR "sars-cov-2" OR sars* OR coronavirus OR corona OR "2019-nCov") | 24.05.: 18,656  27.05.: 18,689 |
|  | **AND Germany**  ("germany" OR "german*" OR "deutsch*") | 24.05.: 3,780  27.05.: 3,788 |
|  | **Filter: from 2018 – 2024**  PUBYEAR > 2017 AND PUBYEAR < 2025 | 24.05.: 3,559  27.05.: 3,566 |
|  | **Without reviews** | 24.05.: 2,421  **27.05.: 2,426** |

| **Embase 27.05.2024** |  |
| --- | --- |
| **#CVD OR #I25 OR #I21 OR #I50 OR #I11 OR #I48 OR #I63 OR #I69 OR #I13 OR #I35 OR #I64 (Title & Abstract)**  **AND Corona (Title, Abstract & Keyword) AND Germany (all fields)**  (((‘cardiovascular disease*’:ab,ti OR ‘cardiovascular disorder*’:ab,ti OR ‘disease of the circulatory system’:ab,ti OR ‘CVD’:ab,ti OR ‘heart disease*’:ab,ti OR ‘vascular disease*’:ab,ti) OR (‘chronic ischemic heart disease*’:ab,ti OR ‘coronary heart disease*’:ab,ti OR ‘atherosclerotic cardiovascular disease*’:ab,ti OR ‘atherosclerotic heart disease*’:ab,ti OR ‘old myocardial infarct*’:ab,ti OR ‘myocardial ischemia‘:ab,ti OR ‘aneurysm of heart’:ab,ti OR ‘ischaemic cardiomyopathy’:ab,ti OR ‘myocardial ischaemia’:ab,ti) OR (’myocardial infarction’:ab,ti OR ‘ST Elevation Myocardial Infarction’:ab,ti OR ‘STEMI’:ab,ti OR ‘NSTEMI’:ab,ti OR ‘cardial infarct*’:ab,ti OR ‘heart attack*’:ab,ti) OR (‘heart insufficienc*’:ab,ti OR ‘heart failure’:ab,ti OR ‘left ventricular failure*’:ab,ti) OR (‘hypertensive heart disease*’:ab,ti) OR (‘atrial flutter’:ab,ti OR ‘atrial fibrillation’:ab,ti) OR (‘cerebral infarct*’:ab,ti OR ‘cerebellar infarct*’:ab,ti OR ‘brain infarct*’:ab,ti OR ‘cerebrovascular infarct*’:ab,ti OR ‘stroke’:ab,ti OR ‘apoplexy’:ab,ti OR ‘cerebrovascular accident’:ab,ti OR ‘CVA’:ab,ti OR ‘vascular accident’:ab,ti) OR (‘cerebrovascular disorder*’:ab,ti OR ‘sequelae of cerebrovascular disease*’:ab,ti OR ‘cerebrovascular disease*’:ab,ti) OR (‘hypertensive heart and renal disease*’:ab,ti OR ‘hypertensive heart disease*’:ab,ti OR ‘hypertensive kidney disease*’:ab,ti OR ‘hypertensive renal disease*’:ab,ti) OR (‘nonrheumatic aortic valve disorder*’:ab,ti OR ‘aortic valve stenosis’:ab,ti OR ‘aortic stenosis’:ab,ti OR ‘aortic valve insufficienc*’:ab,ti OR ‘aortic insufficienc*’:ab,ti OR ‘aortic valve disorder*’:ab,ti OR ‘Aortic Valve Disease’:ab,ti) OR (‘stroke not specified as haemorrhage or infarct*’:ab,ti)) AND (‘pandemic*’:kw,ab,ti OR ‘COVID-19’:kw,ab,ti OR ‘covid*’:kw,ab,ti OR CoV OR ‘sars-cov-2’ OR ‘sars*’:kw,ab,ti OR ‘coronavirus*’:kw,ab,ti OR corona:kw,ab,ti OR ‘2019-nCov’:kw,ab,ti) AND (german* OR germany))  **Filter: from 2018 – 2024** | **1,769** |

Table S 18: Search result for cancer - deduplication with Endnote & Covidence

| **Date** | **Database** | **Results** |
| --- | --- | --- |
| 29.01.2024 | PubMed | 1,302 |
| 29.01.2024 | Scopus | 321 |
| 06.02.2024 | Cochrane library | 30 |
| 02.02.2024 | Web of Science | 1,438 |
| 29.01.2024 | Embase | 390 |
| 12.02.2024 | PubMed German | 303 |
| 19.02.2024 | Scopus German | 104 |
| 28.02.2024 | Cochrane library German | 8 |
| 28.02.2024 | Web of Science German | 386 |
| 28.02.2024 | Embase German | 115 |
|  | **Total** | **4,397** |
| Deduplication | | 1,991 |

Table S 19: Search result for CVD - deduplication with Endnote & Covidence

| **Date** | **Database** | **Results** | **Total**  **results**  **before** | **Duplicates** | **Total results**  **after** | **Covidence** |
| --- | --- | --- | --- | --- | --- | --- |
| 16.05.24 | PubMed | 1,935 | 3,610 | Endnote: 1110  Covidence: 0 | 2,500 | 16.05: +2,500 |
|  | WoS | 1,267 |  |  |  |  |
|  | Cochrane | 408 |  |  |  |  |
| 27.05.24 | PubMed | 1,942  Author, Year, Title: 1,936  Title: 1,936  Author, Year: 1,936 | **7,820** | Endnote: 6 | 7,741 |  |
|  | WoS | 1,275  Author, Year, Title: 1,272  Title: 1,269  Author, Year 1,264 |  | Endnote: 11 |  |  |
|  | Cochrane | 408  Author, Year, Title: 395  Title: 392  Author, Year: 391 |  | Endnote: 17 |  |  |
|  | Scopus | 2,426  Author, Year, Title: 2,422  Title: 2,419  Author, Year: 2,413 |  | Endnote: 13 |  |  |
|  | Embase | 1,769  Author, Year, Title: 1,758  Title: 1,742  Author, Year: 1,737 |  | Endnote: 32 |  |  |
|  | PubMed  WoS  Cochrane  Scopus  Embase | 7,741  Author, Year, Title: 5,663  Title: 5,306  Author, Year: 5,207  DOI: 5,180  2018-2024: 5,009 | 7,741 | Endnote: 2,732 | 5,009 |  |
| 16.05 + 27.05.24 | PubMed  WoS  Cochrane  Scopus  Embase | 2,500 + 5,009  keep older result: 2,500 + 2,897 | 2,500  +5,009  = 7,509 | Endnote: 2,112 | 2,500 +2,897  = 5,397 | 27.05,: +2,897 |
| 27.05.24 |  |  |  | Covidence: 361 | 2,536 | 2,500 + 2,536  = 5,036 |
| 21.05.-18.06.24 |  |  |  | Covidence  manually: 55 |  | 4,981 |
|  |  |  |  |  |  |  |
| **Total** | Search from 16.05: |  | 3,610 | 1,110 | 2500 | 2,500 |
|  | Search from 16.+27.5.: |  | 7,820 | 2,784 | 2500  + 2536 | 5,036 |
|  | Final results  18.06.: |  | 11,430 | 6,449 | **4,981** | **4,981** |

Table S 20: Excluded studies from the full text screening cancer (breast, lung, pancreatitis)

|  | First author | year | title | Reason for exclusion |
| --- | --- | --- | --- | --- |
| 1 | Aeppli et al. | 2020 | Impact of COVID-19 pandemic on treatment patterns in metastatic clear cell renal cell carcinoma. | Wrong setting |
| 2 | Aksakal et al. | 2022 | Effects of the COVID-19 Pandemic on Health Care Services in the Field of Cancer Treatment and Prevention: a qualitative Survey of Care Users | Wrong outcome |
| 3 | Akula | 2022 | Cancer therapy and treatments during COVID-19 era. | Wrong outcome |
| 4 | Alkatout et al. | 2021 | Has COVID-19 Affected Cancer Screening Programs? A Systematic Review. | Wrong study design |
| 5 | AslanKayıran  t al. | 2020 | Approach to skin malignancies in COVID-19 pandemic days. | Wrong setting not Germany |
| 6 | Balakrishnan | 2020 | Delivery of hepato-pancreato-biliary surgery during the COVID-19 pandemic: an European-African Hepato-Pancreato-Biliary Association (E-AHPBA) cross-sectional survey. | Wrong setting no separated data for Germany |
| 7 | Barcellini | 2022 | A year of pandemic for European particle radiotherapy: A survey on behalf of EPTN working group. | Wrong outcome |
| 8 | Bäßler | 2022 | Barriers to access cancer screening and treatment services in Germany | Only poster |
| 9 | Beatrici | 2023 | Clinical stage and grade migration of localized prostate cancer at diagnosis during the past decade. | Wrong setting |
| 10 | Calabrò | 2020 | Challenges in lung cancer therapy during the COVID-19 pandemic. | Wrong outcome |
| 11 | Chazan | 2020 | Impact of COVID-19 on cancer service delivery: results from an international survey of oncology clinicians. | Aggregated data for Europe |
| 12 | Chazan | 2020 | Impact of COVID-19 on cancer service delivery: a follow-up international survey of oncology clinicians | Aggregated data for Europe |
| 13 | Sundar et al. | 2020 | Impact of the covid pandemic on gynaecological cancer surgery – results from the covidsurg gynaecological cancer international study | Wrong indication |
| 14 | Glasby et al. | 2021 | Effect of COVID-19 pandemic lockdowns on planned cancer surgery for 15 tumour types in 61 countries: an international, prospective, cohort study | Wrong outcomes |
| 15 | Desideri et al. | 2020 | Caring for older patients with cancer during the COVID-19 pandemic: A Young International Society of Geriatric Oncology (SIOG) global perspective. | Wrong indication  Leucemia |
| 16 | DeSouza et al. | 2023 | Impact of COVID-19 on 1-Year Survival Outcomes in Hepatocellular Carcinoma: A Multicenter Cohort Study. | Wrong setting |
| 17 | Doege et al. | 2021 | Restrictions in oncological care during the first and second wave of the COVID-19 pandemic in Germany-a prospective panel among 18 comprehensive cancer centers | Wrong outcome |
| 18 | Doege et al. | 2021 | Global change of surgical and oncological clinical practice in urology during early COVID-19 pandemic. | Only conference abstract |
| 19 | Dotzauer et al. | 2021 | Global change of surgical and oncological clinical practice in urology during early COVID-19 pandemic. | Wrong setting/ cancer type |
| 20 | Fauser et al. | 2023 | [Impact of the SARS-CoV-2 Pandemic on the Utilization of Cancer Rehabilitation: a Difference-in-Differences Analysis]. | Wrong setting rehabilitation |
| 21 | Felberbaum et al. | 2021 | Preparing for a pandemic | Wrong outcome |
| 22 | Fotopoulou et al. | 2022 | Outcomes of gynecologic cancer surgery during the COVID-19 pandemic: an international, multicenter, prospective CovidSurg-Gynecologic Oncology Cancer study | Wrong setting no separate data for Germany |
| 23 | Freitas et al. | 2022 | Impact of COVID-19 pandemic on breast cancer neoadjuvant therapy: A retrospective institutional analysis | Wrong setting no German Data reported |
| 24 | Gaisser et al. | 2022 | How the corona pandemic affects cancer patients: Results from a cross-sectional survey in Germany | Wrong outcomes |
| 25 | Gambichler | 2020 | Cancer and Immune Checkpoint Inhibitor Treatment in the Era of SARS-CoV-2 Infection. | Wrong outcome |
| 26 | Garg et al. | 2020 | Discordance of COVID-19 guidelines for patients with cancer: A systematic review | Wrong study design / SR |
| 27 | Gasparri et al. | 2020 | Changes in breast cancer management during the Corona Virus Disease 19 pandemic: An international survey of the European Breast Cancer Research Association of Surgical Trialists (EUBREAST). | Wrong setting no German Data reported |
| 28 | Giammarile et al. | 2021 | Changes in the global impact of COVID-19 on nuclear medicine departments during 2020: an international follow-up survey | Wrong setting no German Data reported |
| 29 | Gremke et al. | 2023 | EPH134 Prevalence of and Associated Factors for Therapy Delay Due to COVID-19 Pandemic Among Breast Cancer Patients in Europe | Wrong setting no German Data reported |
| 30 | Gschnell et al. | 2021 | COVID-19-Lockdown Impacts Medical Care - A Retrospective Analysis of the First Wave at a University Outpatient Clinic in Spring 2020 | Wrong indication |
| 31 | Hekmat et al. | 2020 | COVID-19 guidelines for the prioritization of operations for malignant thoracic diseases | Wrong study design/ guideline |
| 32 | Hölzel et al. | 2022 | Estimation of the Risk of Progression of Breast Cancer After the COVID-19 Lockdown | Wrong outcome modelling |
| 33 | Kaiseret al. | 2022 | Consequences of the Corona crisis on outpatient oncological care - a qualitative study among nurses and medical assistants. | Wrong study design |
| 34 | Kirchberg et al. | 2021 | Influence of the First Wave of the COVID-19 Pandemic on Cancer Care in a German Comprehensive Cancer Center. | Wrong outcome |
| 35 | Klein et al. | 2023 | COVID-19: setback for colorectal cancer screening | Wrong outcome |
| 36 | Lohmiller et al. | 2021 | Acceptance of Psycho-Oncological Counseling Formats in a Cancer Counseling Center during the COVID-19 Pandemic: An Exploratory Care Study. | Wrong study design |
| 37 | Lugnier et al. | 2022 | Cross-sectoral colorectal cancer care in Germany in the context of the COVID-19 pandemic and scarcity of resources | Wrong outcome |
| 38 | Marschner et al. | 2020 | SarsCov2 pandemic and patients with metastatic cancers in Germany-Data from four prospective cancer registries | Wrong outcome |
| 39 | Mathelin | 2021 | Breast Cancer Management During the COVID-19 Pandemic: The Senologic International Society Survey | Wrong outcomes |
| 40 | Muñoz-Martínez et al. | 2021 | Assessing the impact of COVID-19 on liver cancer management (CERO-19) | Wrong setting no German data reported |
| 41 | Muschol et al. | 2023 | COVID-19 related decline in cancer screenings most pronounced for elderly patients and women in Germany: a claims data analysis. | Wrong outcomes |
| 42 | Nasser et al. | 2021 | Implications of the COVID-19 pandemic: Results of a German survey on patient care and clinical trials in gynecological oncology (monitor-17 survey) | Wrong outcomes |
| 43 | Nebelung et al. | 2023 | Impact of the COVID-19 pandemic on therapeutic interventional oncology procedures and diagnostic CT/MRI examinations at a German university hospital | Wrong outcomes/ modelling |
| 44 | Nevermann et al. | 2020 | COVID-19 pandemic: implications on the surgical treatment of gastrointestinal and hepatopancreatobiliary tumours in Europe. | Aggregated data for Europe |
| 45 | Oba et al. | 2020 | Global Survey on Pancreatic Surgery During the COVID-19 Pandemic | Wrong outcomes |
| 46 | Onesti et al. | 2020 | Oncological care organisation during COVID-19 outbreak | Aggregated data for Europe |
| 47 | Ozturk et al. | 2022 | COVID-19 outbreak may increase mortality rates in genitourinary cancers | Aggregated data for Europe |
| 48 | Pergolini et al. | 2021 | Effects of COVID-19 Pandemic on the Treatment of Pancreatic Cancer: A Perspective from Central Europe | Wrong setting no German data reported |
| 49 | Pfister et al. | 2021 | [COVID-19 in urology: Influence of the pandemic on telemedicine, education and surgery]. | Wrong outcome |
| 50 | Prager et al. | 2021 | The impact of COVID-19 on daily practice patterns in the third-line setting for patients with metastatic colorectal cancer: Results of a real-world survey | Wrong setting |
| 51 | Reinacher-Schick et al. | 2023 | Effects of the Pandemic on the Care of Patients With Colorectal Cancer | Wrong study design |
| 52 | Riemann et al. | 2021 | Colon cancer screening in times of COVID-19 | Wrong outcomes |
| 53 | Röhnsch et al. | 2023 | Coping with subjectively experienced coronavirus risks: perspectives of young, chronically ill adults | Wrong outcomes |
| 54 | Santic et al. | 2023 | COVID-19 and its Consequences: Isolated Increase in Patients treated with Radiation Oncology only for Prostate Cancer during the Pandemic | Wrong outcomes |
| 55 | Scheidt-Nave | 2021 | Care for the chronically ill in Germany - The challenges during the COVID-19 pandemic | Wrong study design |
| 56 | Schneider et al. | 2023 | The impact of the COVID-19 pandemic restrictions on the health care utilization of cancer patients. | Wrong outcomes |
| 57 | Schultz et al. | 2023 | Impact of COVID-19 pandemic conditions on disease progression and prognosis of patients with malignant melanoma | Wrong outcomes |
| 58 | Seretis et al. | 2022 | A Meta-Analysis on the Impact of the COVID-19 Pandemic on Cutaneous Melanoma Diagnosis in Europe | Wrong study design |
| 59 | Sommerlatte et al. | 2021 | Allocating resources in cancer care during Sars-CoV-2 pandemic. Preliminary findings and ethical analysis from a qualitative interview study with oncologists | Wrong study design |
| 60 | Sommerlatte et al. | 2022 | Allocating resources in cancer care during pandemic. Findings from a qualitative interview study with oncologists and ethical analysis | Wrong study design |
| 61 | Staehler etla. | 2021 | Counterbalancing COVID-19 with Cancer Surveillance and Therapy: A Survey of Patients with Renal Cell Carcinoma | Wrong setting |
| 62 | Staehler et al. | 2021 | COVID-19 and financial toxicity in patients with renal cell carcinoma | Wrong outcomes |
| 63 | Stöss et al. | 2021 | Impact of the COVID-19 Pandemic on Surgical Oncology in Europe: Results of a European Survey | Wrong setting |
| 64 | VanDam et al. | 2022 | The impact of the SARS-COV-2 pandemic on the quality of breast cancer care in EUSOMA-certified breast centres | Wrong setting |
| 65 | vandeHaar et al | 2022 | Caring for patients with cancer in the COVID-19 era | Wrong outcomes |
| 66 | Vigliar et al. | 2022 | COVID-19 pandemic impact on cytopathology practice in the post-lockdown period: An international, multicenter study | Wrong outcomes |
| 67 | VogelMME | 2022 | Coronavirus disease 2019 and radiation oncology-survey on the impact of the severe acute respiratory syndrome coronavirus 2 pandemic on health care professionals in radiation oncology | Wrong outcomes |
| 68 | Vu et al. | 2021 | Nationwide Survey of German Outpatient Gynecologic Oncology Practices during the Coronavirus Disease 2019 Pandemic: Reactions to the First Wave and Future Perspectives. | Wrong patient population |
| 69 | Wakefield | 2020 | Initial Impact and Operational Response of Radiation Oncology Practices to the COVID-19 Pandemic in the United States, Europe, and Latin America. | Wrong setting |
| 70 | Zattoni | 2021 | Has the COVID-19 outbreak changed the way we are treating prostate cancer? An EAU - YAU Prostate Cancer Working Group multi-institutional study. | Wrong setting |

Table S 21: Excluded studies from the full text screening cancer (breast, lung, pancreatitis)

|  | First author | year | title | Reason for exclusion |
| --- | --- | --- | --- | --- |
| 1 | Al Kasab et al. | 2020 | International experience of mechanical thrombectomy during the COVID-19 pandemic: insights from STAR and ENRG | Wrong outcome |
| 2 | Ali et al. | 2023 | Recovery From the Impact of COVID-19 on Treatment Times and Clinical Outcomes of Patients With ST-Segment Elevation Myocardial Infarction: An Interim Analysis | Wrong setting |
| 3 | Alicandro et al. | 2023 | A comprehensive analysis of all-cause and cause-specific excess deaths in 30 countries during 2020 | Wrong outcome |
| 4 | Altersberger et al. | 2021 | Maintenance of Acute Stroke Care Service During the COVID-19 Pandemic Lockdown | Wrong indication: I63 ischemic stroke |
| 5 | Andress et al. | 2022 | Deferral of non-emergency cardiac procedures is associated with increased early emergency cardiovascular hospitalizations | Wrong indication: various CVD aggregated |
| 6 | Andreß et al. | 2023 | Predictors of worse outcome after postponing non-emergency cardiac interventions during the COVID-19 pandemic | Wrong indication: various CVD aggregated |
| 7 | Andress et al. | 2024 | Deferral of non-emergency cardiac interventions is associated with increased emergency hospitalizations up to 24 months post-procedure | Wrong indication: various CVD aggregated |
| 8 | Araiza-Garaygor et al. | 2021 | Impact of the COVID-19 pandemic on hospitalizations for acute coronary syndromes: a multinational study | Wrong setting |
| 9 | Beckmann et al. | 2023 | German Heart Surgery Report 2022: The Annual Updated Registry of the German Society for Thoracic and Cardiovascular Surgery | Wrong indication |
| 10 | Beckmann et al. | 2022 | German Heart Surgery Report 2021: The Annual Updated Registry of the German Society for Thoracic and Cardiovascular Surgery | Wrong indication |
| 11 | Beckmann et al. | 2021 | German Heart Surgery Report 2020: The Annual Updated Registry of the German Society for Thoracic and Cardiovascular Surgery | Wrong indication |
| 12 | Behrendt et al. | 2021 | How Does SARS-CoV-2 Infection Affect Survival of Emergency Cardiovascular Patients? A Cohort Study From a German Insurance Claims Database | Wrong indication: I21 STEMI & NSTEMI, stroke |
| 13 | Belau et al. | 2024 | Trends in stroke severity at hospital admission and rehabilitation discharge before and during the COVID-19 pandemic in Hesse, Germany: a register-based study | Wrong indication: I63 ischemic stroke |
| 14 | Ben-Haim et al. | 2021 | The Pandemic's impacts on patients without Covid-19 on multidisciplinary aspects in emergency medicine care | Wrong setting |
| 15 | Bersano et al. | 2020 | Stroke care during the COVID-19 pandemic: experience from three large European countries | Wrong indication: I63 ischemic stroke |
| 16 | Bette et al. | 2023 | Treatment of acute mesenteric ischemia between 2010 and 2020 - a German nation-wide study | Wrong indication |
| 17 | Bilionis et al. | 2023 | A Survey on Public Data Sets Related to Chronic Diseases | Wrong setting |
| 18 | Bollmann et al. | 2020 | In-hospital mortality in heart failure in Germany during the Covid-19 pandemic | Wrong indication: heart failure aggregated |
| 19 | Bollmann et al. | 2021 | Cumulative hospitalization deficit for cardiovascular disorders in Germany during the COVID-19 pandemic: insights from the German-wide Helios hospital network | Wrong indication: heart failure aggregated |
| 20 | Bollmann et al. | 2021 | Utilization of in- and outpatient hospital care in Germany during the Covid-19 pandemic insights from the German-wide Helios hospital network | Wrong outcomes |
| 21 | Boriani et al. | 2023 | Length of hospital stay for elective electrophysiological procedures: a survey from the European Heart Rhythm Association | Wrong outcomes |
| 22 | Brunner et al. | 2021 | Acute coronary syndrome-related hospital admissions during and after lockdown in Southern Germany | Wrong indication |
| 23 | Brunssen et al. | 2022 | Stroke care during the COVID-19 pandemic: Case numbers, treatments, and mortality in two large German stroke registries | Wrong indication: I63 cerebral infarction, I64 stroke, not specified as hemorrhage or infarction |
| 24 | Cârstea et al. | 2022 | Profile of Patients with Cardiovascular Diseases during the Pandemic in a Cardiology Clinic of a COVID-19 Support Hospital | Wrong setting |
| 25 | Choi et al. | 2021 | Efficacy of a four-tier infection response system in the emergency department during the coronavirus disease-2019 outbreak | Wrong setting |
| 26 | Chourasia | 2022 | Admissions to the Emergency Department Due to Atrial Fibrillation/Atrial Flutter Incidents during the Third Wave of COVID-19 Pandemic | Wrong setting |
| 27 | Chowdhury | 2021 | Management of acute ischemic stroke in the interventional neuroradiology suite during the COVID-19 pandemic: A global survey | Wrong outcomes |
| 28 | Clifford et al. | 2021 | Delays in ST-Elevation Myocardial Infarction Care During the COVID-19 Lockdown: An Observational Study | Wrong setting |
| 29 | De Luca et al. | 2022 | COVID-19 pandemic, mechanical reperfusion and 30-day mortality in ST elevation myocardial infarction | Wrong outcomes |
| 30 | De Luca et al. | 2020 | Impact of COVID-19 Pandemic on Mechanical Reperfusion for Patients With STEMI | Wrong outcomes |
| 31 | De Sousa et al. | 2020 | Maintaining stroke care in Europe during the COVID-19 pandemic: Results from an international survey of stroke professionals and practice recommendations from the European Stroke Organisation | Wrong outcomes |
| 32 | Dengler et al. | 2022 | Changes in nationwide in-hospital stroke care during the first four waves of COVID-19 in Germany | Wrong indication: I63 ischemic stroke |
| 33 | Dorr et al. | 2022 | Heart failure hospitalizations in Germany 2000-2020: Long-term temporal trends and impact of COVID-19 in a nationwide complete census of in-patient routine data | Wrong patient population |
| 34 | Dreger et al. | 2020 | Acute myocardial infarction admissions in Berlin during the COVID-19 pandemic | Wrong indication: I21 acute myocardial infarction |
| 35 | Du et al. | 2024 | Outpatient Health Service Utilization Among Adults with Diabetes, Hypertension and Cardiovascular Disease During the COVID-19 Pandemic - Results of Population-Based Surveys in Germany from 2019 to 2021 | Wrong indication: I21 acute myocardial infarction, stroke |
| 36 | Eckner et al. | 2021 | Differences in the Treatment of Acute Coronary Syndrome in the Pre-COVID and COVID Era: An Analysis from Two German High-Volume Centers | Wrong indication: I21 STEMI & NSTEMI, stroke |
| 37 | El Ouaddi et al. | 2022 | Impact of COVID-19 on mechanical complications in ST elevation myocardial infarction | Wrong setting |
| 38 | Equiza et al. | 2022 | Impact on functional outcome of an adaptive Stroke Unit based system of care for patients undergoing endovascular treatment during pandemic times | Wrong setting |
| 39 | Erdur et al. | 2021 | Stroke Admissions, Stroke Severity, and Treatment Rates in Urban and Rural Areas During the COVID-19 Pandemic | Wrong indication: I63 ischemic stroke |
| 40 | Eun et al. | 2022 | Effects of an Infection Control Protocol for Coronavirus Disease in Emergency Mechanical Thrombectomy | Wrong setting |
| 41 | Falter et al. | 2022 | Declining Numbers of Neurosurgical Emergencies at a German University Medical Center during the Coronavirus Lockdown | Wrong indication |
| 42 | Felbel et al. | 2022 | Deferral of Non-Emergency Cardiovascular Interventions Triggers Increased Cardiac Emergency Admissions-Analysis of the COVID-19 Related Lockdown | Wrong indication |
| 43 | Ferhatbegovic et al. | 2023 | Characteristics of acute coronary syndrome during COVID 19 pandemic | Wrong setting |
| 44 | Fiehler et al. | 2020 | COVID-19 and neurointerventional service worldwide: a survey of the European Society of Minimally Invasive Neurological Therapy (ESMINT), the Society of NeuroInterventional Surgery (SNIS), the Sociedad Iberolatinoamericana de Neuroradiologia Diagnostica y Terapeutica (SILAN), the Society of Vascular and Interventional Neurology (SVIN), and the World Federation of Interventional and Therapeutic Neuroradiology (WFITN) | Wrong outcomes |
| 45 | Fluck et al. | 2022 | Does the length of stay in hospital affect healthcare outcomes of patients without COVID-19 who were admitted during the pandemic? A retrospective monocentric study | Wrong setting |
| 46 | Gaede et al. | 2021 | Impact of COVID-19 lockdown on the procedural and intra-hospital outcome of STEMI patients | Wrong indication: I21 STEMI |
| 47 | Gao et al. | 2022 | Reconsidering treatment guidelines for acute myocardial infarction during the COVID-19 pandemic | Wrong setting |
| 48 | Gaßmann et al. | 2022 | Stroke Care Performance measures during the COVID-19 pandemic - over the waves | Wrong indication: I63 ischemic stroke |
| 49 | Gawalko et al. | 2021 | The European TeleCheck-AF project on remote app-based management of atrial fibrillation during the COVID-19 pandemic: centre and patient experiences | Wrong outcomes |
| 50 | Gellert et al. | 2023 | Hospital admissions and deaths due to acute cardiovascular events during the COVID-19 pandemic in residents of long-term care facilities | Wrong indication: I21 STEMI & NSTEMI, I63 ischemic stroke |
| 51 | Gheewala et al. | 2023 | Decompressive Surgery in the Treatment of Acute Ischemic Stroke during the First Four Waves of the COVID-19 Pandemic in Germany: A Nationwide Observational Cohort Study | Wrong indication: I63 ischemic stroke |
| 52 | Gitt et al. | 2020 | Collateral damage of COVID-19-lockdown in Germany: decline of NSTE-ACS admissions | Wrong indication: I21 STEMI & NSTEMi |
| 53 | Gori et al. | 2020 | Perspective: cardiovascular disease and the Covid-19 pandemic | Wrong study design |
| 54 | Grad et al. | 2022 | Scars of stroke care emerge as COVID-19 shifts to an endemic in many countries | Wrong indication: stroke |
| 55 | Griewing et al. | 2022 | Chronological Development of Cardiovascular Disease in Times of COVID-19: A Retrospective Analysis of Hospitalized Diseases of the Circulatory System and COVID-19 Patients of a German University Hospital | Wrong patient population |
| 56 | Großmann et al. | 2024 | Left Ventricular Reconstruction after Dor-Sailing Close to the Wind? | Wrong outcomes |
| 57 | Hagebusch et al. | 2020 | Decline in emergency medical service missions during the COVID-19 pandemic: results from the fifth largest city in Germany | Wrong indication: acute coronary syndrome incl. STEMI |
| 58 | Hahn et al. | 2023 | Association of Delirium Incidence with Visitation Restrictions due to COVID-19 Pandemic in Patients with Acute Cerebrovascular Disease in a Stroke-Unit Setting: A Retrospective Cohort Study | Wrong indication: I63 ischemic stroke |
| 59 | Hajdu et al. | 2020 | Acute Stroke Management During the COVID-19 Pandemic Does Confinement Impact Eligibility for Endovascular Therapy? | Wrong outcomes |
| 60 | Hecht et al. | 2020 | Need for ensuring care for neuro-emergencies-lessons learned from the COVID-19 pandemic | Wrong outcomes |
| 61 | Heidenreich et al. | 2023 | Impact of the COVID-19 pandemic on aortic valve replacement procedures in Germany | Wrong outcomes |
| 62 | Howley et al. | 2021 | Trends in emergency department use by older people during the COVID-19 pandemic | Wrong setting |
| 63 | Hoyer et al. | 2020 | Acute Stroke in Times of the COVID-19 Pandemic A Multicenter Study | Wrong indication: I63 ischemic stroke |
| 64 | Hoyer et al. | 2020 | Changes in Demographic and Diagnostic Spectra of Patients with Neurological Symptoms Presenting to an Emergency Department During the COVID-19 Pandemic: A Retrospective Cohort Study | Wrong indication: I63 ischemic stroke |
| 65 | Ikenberg et al. | 2020 | Code Stroke Patient Referral by Emergency Medical Services During the Public COVID-19 Pandemic Lockdown | Wrong indication: I63 ischemic stroke |
| 66 | Jansen et al. | 2021 | Consequences of COVID-19 pandemic lockdown on emergency and stroke care in a German tertiary stroke center | Wrong indication: I63 ischemic stroke |
| 67 | Kämpfer et al. | 2021 | Reorganizing stroke and neurological intensive care during the COVID-19 pandemic in Germany | Wrong indication: I63 ischemic stroke |
| 68 | Kapsner et al. | 2021 | Reduced Rate of Inpatient Hospital Admissions in 18 German University Hospitals During the COVID-19 Lockdown | Wrong indication: I21 myocardial infarction |
| 69 | Keramida et al. | 2020 | Distribution, infrastructure, and expertise of heart failure and cardio-oncology clinics in a developing network: temporal evolution and challenges during the coronavirus disease 2019 pandemic | Wrong setting |
| 70 | Keskin et al. | 2021 | Should we postpone elective cardiovascular procedures and percutaneous coronary interventions during the covid-19 pandemic? | Wrong setting |
| 71 | Kin et al. | 2022 | Differential Impact of the COVID-19 Pandemic on Health Care Utilization Disruption for Community-Dwelling Individuals With and Without Acquired Brain Injury | Wrong setting |
| 72 | Klingner et al. | 2020 | Effectiveness, efficiency and safety of stroke telemedicine in times of the coronavirus pandemic The "case" Thuringia | Wrong indication: I63 ischemic stroke |
| 73 | Koh et al. | 2022 | Impact of COVID-19 pandemic on STEMI thrombolysis and Emergency Department's performance in a non-PCI capable tertiary hospital | Wrong setting |
| 74 | König et al. | 2020 | In-hospital care in acute heart failure during the COVID-19 pandemic: insights from the German-wide Helios hospital network | Wrong indication: heart failure aggregated |
| 75 | König et al. | 2021 | Hospitalization deficit of in- and outpatient cases with cardiovascular diseases and utilization of cardiological interventions during the COVID-19 pandemic: Insights from the German-wide helios hospital network | Wrong indication: heart failure aggregated |
| 76 | Korvink et al. | 2022 | A Novel Approach to Attribute Responsible Physicians Using Inpatient Claims | Wrong setting |
| 77 | Krieger et al. | 2022 | Evaluating the impact of the covid-19 pandemic on mortality after myocardial infarctions hospitalization in germany | Wrong indication: I21 acute myocardial infarction |
| 78 | Lechner et al. | 2022 | Impact of COVID-19 pandemic restrictions on ST-elevation myocardial infarction: A cardiac magnetic resonance imaging study | Wrong indication: I21 STEMI |
| 79 | Lee et al. | 2021 | Impact of covid-19 lockdown on adult cardiac surgery in a public hospital | Wrong indication |
| 80 | Lintas et al. | 2022 | Non-ischemic neurovascular emergencies at a supra-regional medical center during the SARS-CoV2-pandemia | Wrong indication |
| 81 | Lopes et al. | 2024 | The impact of the COVID-19 pandemic on the management of acute coronary syndrome: a retrospective cohort study | Wrong setting |
| 82 | Lübcke et al. | 2024 | Short Term Outcomes and Treatment Intensity of Major Cardiovascular Emergencies During the COVID-19 Pandemic in Germany | Wrong indication: I21 STEMI & NSTEMI |
| 83 | Macherey et al. | 2021 | Impact of respiratory infectious epidemics on STEMI incidence and care | Wrong indication: Ire STEMI |
| 84 | Maehl et al. | 2021 | The Impact of the COVID-19 Pandemic on Avoidance of Health Care, Symptom Severity, and Mental Well-Being in Patients With Coronary Artery Disease | Wrong indication: chronic ischemic heart disease aggregated |
| 85 | Malhi et al. | 2022 | Care and Outcomes of ST-Segment Elevation Myocardial Infarction Across Multiple COVID-19 Waves | Wrong setting |
| 86 | Mariani et al. | 2021 | Telemonitoring and Care Program for Left Ventricular Assist Device Patients During COVID-19 Outbreak: A European Experience | Wrong indication |
| 87 | Martsevich et al. | 2022 | Effects of the COVID-19 Pandemic on Treatment Adherence in Patients with Chronic Heart Failure | Wrong setting |
| 88 | Mathew et al. | 2021 | Cardiac arrhythmias in patients with SARS-CoV‑2 infection and effects of the lockdown on invasive rhythmological therapv | Wrong indication: various CVD aggregated incl. atrial fibrillation/ flutter |
| 89 | Michalowsky et al. | 2021 | Effect of the COVID-19 lockdown on disease recognition and utilisation of healthcare services in the older population in Germany: a cross-sectional study | Wrong indication: chronic ischemic heart disease aggregated |
| 90 | Millán et al. | 2021 | Differential effects of the SARS-CoV-2 pandemic on patients presenting to a neurological emergency room depending on their triage score in an area with low COVID-19 incidence | Wrong outcomes |
| 91 | Mitgutsch et al. | 2022 | Patient autonomy in cardiac inpatient rehabilitation—A COVID-19-specific exploratory trend study | Wrong setting |
| 92 | Nef et al. | 2021 | Impact of the COVID-19 pandemic on cardiovascular mortality and catherization activity during the lockdown in central Germany: an observational study | Wrong indication: I21 STEMI & NSTEMI,  various CVD aggregated |
| 93 | Nguyen et al. | 2022 | Global Impact of the COVID-19 Pandemic on Cerebral Venous Thrombosis and Mortality | Wrong outcomes |
| 94 | Nguyen et al. | 2023 | Global Impact of the COVID-19 Pandemic on Stroke Volumes and Cerebrovascular Events A 1-Year Follow-up | Wrong outcomes |
| 95 | Nogueira et al. | 2021 | Global impact of COVID-19 on stroke care | Wrong outcomes |
| 96 | Nogueira et al. | 2021 | Global Impact of COVID-19 on Stroke Care and IV Thrombolysis | Wrong outcomes |
| 97 | Oettinger et al. | 2023 | COVID-19 pandemic affects STEMI numbers and in-hospital mortality: results of a nationwide analysis in Germany | Wrong indication: I21 STEMI |
| 98 | Ong et al. | 2023 | Clinical and procedural characteristics of patients with acute coronary syndrome during the COVID-19 pandemic 2020 compared to a control group from 2019 | Wrong indication: I21 STEMI |
| 99 | Padjen et al. | 2021 | Acute stroke care during the COVID-19 pandemic | Wrong study design |
| 100 | Pereira et al. | 2022 | Stent-Save a Life international survey on the practice of primary percutaneous coronary intervention during the COVID-19 pandemic | Wrong setting |
| 101 | Perel et al. | 2021 | How the COVID-19 pandemic changed treatment of severe aortic stenosis: a single cardiac center experience | Wrong setting |
| 102 | Pessoa-Amorim et al. | 2020 | Admission of patients with STEMI since the outbreak of the COVID-19 pandemic: a survey by the European Society of Cardiology | Wrong outcomes |
| 103 | Pfaff et al. | 2021 | Neuroradiological emergency consultations during the first year of the COVID-19 pandemic | Wrong outcomes |
| 104 | Pinto-Filho et al. | 2023 | Electrocardiographic findings and prognostic values in patients hospitalised with COVID-19 in the World Heart Federation Global Study What's going on following acute covid-19? Clinical characteristics of patients in an out-patient rehabilitation program Effects of COVID-19 on a mature citizen first responder system in the German district of Gütersloh: an observational study Ventricular arrhythmia burden in patients with implantable cardioverter defibrillator and remote patient monitoring during different time intervals of the COVID-19 pandemic | Wrong indication |
| 105 | Primessnig et al. | 2021 | Increased mortality and worse cardiac outcome of acute myocardial infarction during the early COVID-19 pandemic | Wrong indication: I21 STEMI & NSTEMI |
| 106 | Rattka et al. | 2021 | Outcomes of Patients With ST-Segment Elevation Myocardial Infarction Admitted During COVID-19 Pandemic Lockdown in Germany - Results of a Single Center Prospective Cohort Study | Wrong indication: I21 STEMI |
| 107 | Rattka et al. | 2022 | Outcomes of patients with ST-segment myocardial infarction admitted during the COVID-19 pandemic A prospective, observational study from a tertiary care center in Germany | Wrong indication: I21 STEMI |
| 108 | Rattka et al. | 2020 | 31 days of COVID-19-cardiac events during restriction of public life-a comparative study | Wrong indication: I21 STEMI & NSTEMI |
| 109 | Richter et al. | 2021 | Analysis of Nationwide Stroke Patient Care in Times of COVID-19 Pandemic in Germany | Wrong indication: I63 ischemic stroke |
| 110 | Richter et al. | 2020 | Comparison of stroke care parameters in acute ischemic stroke patients with and without concurrent Covid-19. A Nationwide analysis | Wrong indication: I63 ischemic stroke |
| 111 | Richter et al. | 2022 | A full year of the COVID-19 pandemic with two infection waves and its impact on ischemic stroke patient care in Germany | Wrong indication: I63 ischemic stroke |
| 112 | Richter et al. | 2021 | Acute ischemic stroke care in Germany - further progress from 2016 to 2019 | Wrong patient population |
| 113 | Ronco et al. | 2023 | Impact of COVID-19 on incidence and outcomes of post-infarction mechanical complications in Europe | Wrong indication |
| 114 | Rosati et al. | 2022 | Every cloud has a silver lining: COVID-19 chest-CT screening prevents unnecessary cardiac surgery | Wrong setting |
| 115 | Ryffel et al. | 2023 | Impact of COVID-19 Surge Periods on Clinical Outcomes of Transcatheter Aortic Valve Implantation | Wrong setting |
| 116 | Schäfer et al. | 2023 | Non-utilisation of medical services during the COVID-19 pandemic among persons with chronic diseases | Wrong indication |
| 117 | Scheidt-Nave et al. | 2021 | Care for the chronically ill in Germany - The challenges during the COVID-19 pandemic | Wrong study design |
| 118 | Schlachetzki et al. | 2020 | Decline and Recurrence of Stroke Consultations during the COVID-19 Pandemic Lockdown Parallels Population Activity Levels | Wrong indication: I63 ischemic stroke |
| 119 | Schlachetzki et al. | 2022 | Low stroke incidence in the TEMPiS telestroke network during COVID-19 pandemic: Effect of lockdown on thrombolysis and thrombectomy | Wrong indication: I63 ischemic stroke |
| 120 | Schmitz et al. | 2021 | Impact of COVID-19 pandemic lockdown on myocardial infarction care | Wrong indication: I21 STEMI & NSTEMI |
| 121 | Scholz et al. | 2020 | Impact of COVID-19 outbreak on regional STEMI care in Germany | Wrong indication: I21 STEMI |
| 122 | Schwab et al. | 2022 | Changes in the implantation procedures of pacemakers during the COVID-19 pandemic in Germany | Wrong indication |
| 123 | Schwarz et al. | 2020 | Decline of emergency admissions for cardiovascular and cerebrovascular events after the outbreak of COVID-19 | Wrong indication: I21 STEMI & NSTEMI, I63 ischemic stroke |
| 124 | Sedghi et al. | 2021 | Delayed Comprehensive Stroke Unit Care Attributable to the Evolution of Infection Protection Measures across Two Consecutive Waves of the COVID-19 Pandemic | Wrong outcomes |
| 125 | Sedova et al. | 2023 | The decline in stroke hospitalization due to COVID-19 is unrelated to COVID-19 intensity | Wrong setting |
| 126 | Seiffert et al. | 2020 | Temporal trends in the presentation of cardiovascular and cerebrovascular emergencies during the COVID-19 pandemic in Germany: an analysis of health insurance claims | Wrong indication: I21 STEMI & NSTEMI |
| 127 | Slagman et al. | 2020 | Medical Emergencies During the COVID-19 Pandemic | Wrong indication: I21 myocardial infarction |
| 128 | Sokolski et al. | 2021 | Impact of Coronavirus Disease 2019 (COVID-19) Outbreak on Acute Admissions at the Emergency and Cardiology Departments Across Europe | Wrong outcomes |
| 129 | Soylu et al. | 2021 | Effect of Covid-19 pandemic process on STEMI patients timeline | Wrong setting |
| 130 | Studer et al. | 2021 | COVID-19 Reveals Opportunities for Better Care of Stroke Patients | Wrong outcomes |
| 131 | Sudhan et al. | 2021 | Neurosurgical Outcomes, Protocols, and Resource Management During Lockdown: Early Institutional Experience from One of the World's Largest COVID 19 Hotspots | Wrong setting |
| 132 | Szkudlarek et al. | 2023 | How has the COVID-19 pandemic affected patients with stroke? An emergency department perspective | Wrong setting |
| 133 | Tab et al. | 2021 | Delayed presentation of acute coronary syndrome with mechanical complication during COVID-19 pandemic: a case report | Wrong setting |
| 134 | Tanislav et al. | 2021 | Consultations Decline for Stroke, Transient Ischemic Attack, and Myocardial Infarction during the COVID-19 Pandemic in Germany | Wrong indication: I21 acute myocardial infarction, I63 ischemic stroke,  I64 stroke, not specified as hemorrhage or infarction |
| 135 | Tetzlaff et al. | 2024 | Age-specific and cause-specific mortality contributions to the socioeconomic gap in life expectancy in Germany, 2003-21: an ecological study [Wave riding - 12 months of COVID-19 in a German tertiary care center] | Wrong outcomes |
| 136 | Thevathasan et al. | 2022 | Impact of early readmission to the cardiac ICUon in-hospital mortality and hospital length of stay in 30,942 cardiac patients | Wrong indication |
| 137 | Tiedt et al. | 2020 | Impact of the COVID-19-pandemic on thrombectomy services in Germany | Wrong indication: I63 ischemic stroke |
| 138 | Torres Crigna et al. | 2024 | Global incidence, prevalence, years lived with disability (YLDs), disability-adjusted life-years (DALYs), and healthy life expectancy (HALE) for 371 diseases and injuries in 204 countries and territories and 811 subnational locations, 1990-2021: a systematic analysis for the Global Burden of Disease Study 2021 Endothelin-1 axes in the framework of predictive, preventive and personalised (3P) medicine | Wrong outcomes |
| 139 | Toušek et al. | 2021 | Modified strategies for invasive management of acute coronary syndrome during the covid-19 pandemic | Wrong setting |
| 140 | Ungerer et al. | 2024 | The evolution of acute stroke care in Germany from 2019 to 2021: analysis of nation-wide administrative datasets | Wrong indication: I63 ischemic stroke |
| 141 | Uphaus et al. | 2020 | Stroke Care Within the COVID-19 Pandemic-Increasing Awareness of Transient and Mild Stroke Symptoms Needed | Wrong indication: I63 ischemic stroke |
| 142 | Urbanek et al. | 2020 | Collateral damages in the SARS-CoV-2 pandemia- two cases | Wrong outcomes |
| 143 | Vacanti et al. | 2020 | Reduced rate of admissions for acute coronary syndromes during the COVID-19 pandemic: an observational analysis from a tertiary hospital in Germany | Wrong indication |
| 144 | Vallejo et al. | 2021 | Stroke prevention in patients with atrial fibrillation. Improving protection in the COVID-19 era | Wrong setting |
| 145 | Vollmuth et al. | 2021 | Impact of the coronavirus disease 2019 pandemic on stroke teleconsultations in Germany in the first half of 2020 | Wrong indication: I63 ischemic stroke |
| 146 | Wahler et al. | 2022 | Acute Coronary Heart Syndrom (ACS): Hospitalizations in Germany Before and During COVID-19 Pandemic | Wrong indication: I21 STEMi & NSTEMI |
| 147 | Weckbach et al. | 2021 | Myocardial Inflammation and Dysfunction in COVID-19-Associated Myocardial Injury Global impact of COVID-19 on stroke care | Wrong patient population |
| 148 | Wienbergen et al. | 2021 | Impact of COVID-19 Pandemic on Presentation and Outcome of Consecutive Patients Admitted to Hospital Due to ST-Elevation Myocardial Infarction | Wrong indication: I21 STEMI |
| 149 | Wienhold et al. | 2021 | Teleconsultation for preoperative evaluation during the coronavirus disease 2019 pandemic: A technical and medical feasibility study | Wrong outcomes |
| 150 | Wójcik et al. | 2023 | High in-hospital mortality and prevalence of cardiogenic shock in patients with ST-segment elevation myocardial infarction and concomitant COVID-19 | Wrong setting |
| 151 | Yavagal et al. | 2021 | International Survey of Mechanical Thrombectomy Stroke Systems of Care During COVID-19 Pandemic | Wrong indication: I63 ischemic stroke |
| 152 | Zeymer et al. | 2023 | Effects of the COVID-19 pandemic on acute coronary syndromes in Germany during the first wave: the COVID-19 collateral damage study | Wrong indication: I21 STEMi & NSTEMI |
| 153 | Zeymer et al. | 2021 | COVID-19 pandemic Effects on clinical care of cardiovascular patients in spring 2020 | Wrong study design |
| 154 | Zoghbi et al. | 2020 | Multimodality Cardiovascular Imaging in the Midst of the COVID-19 Pandemic: Ramping Up Safely to a New Normal | Wrong setting |
| 155 | Zylla et al. | 2024 | Global burden of 288 causes of death and life expectancy decomposition in 204 countries and territories and 811 subnational locations, 1990-2021: a systematic analysis for the Global Burden of Disease Study 2021 Predictors and Prognostic Implications of Cardiac Arrhythmias in Patients Hospitalized for COVID-19 | Wrong outcomes |
